# *fruitless* is essential for male *Aedes aegypti* mosquito attraction to female flight sounds

**DOI:** 10.1371/journal.pbio.3004041

**Published:** 2026-10-27

**Authors:** YuMin M. Loh, Alberto J. Thornton, WenWei Loh, Tai-Ting Lee, Wan-Tze Chen, Yifeng Y. J. Xu, Jia Xin Yap, Yuichiro Tsuchiya, Daniel F. Eberl, Matthew P. Su, Azusa Kamikouchi

**Affiliations:** 1 Graduate School of Science, Nagoya University, Nagoya, Japan; 2 Institute of Transformative Bio-Molecules (WPI-ITbM), Nagoya University, Nagoya, Japan; 3 Department of Biology, University of Iowa, Iowa City, Iowa, United States of America; 4 Institute for Advanced Research, Nagoya University, Nagoya, Japan; University of Michigan, UNITED STATES OF AMERICA

## Abstract

Male mosquitoes locate females through strong and innate attraction to female flight sounds, the basis of mating in the world’s deadliest animal. In contrast, females do not show any sound attraction behaviors. Despite being documented for almost a century, the molecular underpinnings of these behavioral dimorphisms remain unknown. Here, we found that by disrupting the male-specific isoform of *fruitless* (referred to as *fruM*) in *Aedes aegypti*, *fruM* mutant males show complete abolition of sound attraction behaviors, resulting in failure of mutant males to locate females for mating. We identified strong *fruitless* expression in control male ears, with the loss of FruM severely disrupting mutant male ability to amplify female flight sounds. *fruM* mutant male ears also propagate frequency-specific, sound-evoked electrical signals at about one-sixth the magnitude of control males, significantly reducing the amount of auditory information relayed to the brain. *fruitless* thus plays a significant role in determining proper male peripheral hearing function underlying mating. Transcriptomic analyses of mosquito ears identified essential hearing genes under the transcriptional influence of FruM *via* direct or indirect actions, thus shaping male-specific hearing cellular machineries that support male attraction to female flight sounds. Our work demonstrates that FruM commands male-specific hearing behaviors involved in mating by directly controlling the earliest stages of auditory organ performance, sound amplification and acoustic signal detection. Our findings show that FruM is essential for male-specific reproductive behaviors in mosquitoes.

## Abstract 2

Male *Aedes aegypti* mosquitoes locate females by listening for their flight sounds. This study shows that disruption of the male specific isoform of *fruitless*, a key sex differentiation gene, alters the male hearing system and suppresses responses to female flight sounds.

## Introduction

Sexual dimorphisms in insect behaviors arise from the need to divide reproductive tasks, which in turn require corresponding dimorphisms in relevant sensory organs involved in the processing of sensory information [1,2]. In disease-transmitting mosquitoes, males form male-dominated mating swarms at dusk and show robust attraction to female flight sounds (‘phonotaxis’) to locate females [3,4]. Females neither swarm nor exhibit phonotaxis, but seek hosts and oviposit [3,5–7].

Mosquito ears are composed of a flagellum coupled with a Johnston’s Organ (JO) housed in the second antennal segment, the pedicel [8]. Unlike other insect species, but like vertebrate ears, mosquito JOs are innervated by auditory efferents descending from the central nervous system (CNS) [9]. Sexual dimorphisms in mosquito ears are apparent at various levels: from the anatomical structure of the flagellum to the number and properties of JO neurons to the localization of auditory efferent terminals, these properties collectively shape dimorphisms in peripheral hearing function [10–12]. Understanding these anatomical and functional dimorphisms represents the first step towards understanding sex-specific hearing behaviors in mosquitoes.

In insects, sex-specific somatic cell proliferation and neuronal differentiation are differentially regulated by *doublesex* (*dsx*) and *fruitless* (*fru*), respectively [13,14]. The former is crucial in shaping sex-specific morphological structures while the latter determines sexual dimorphisms in behaviors [15,16]. Both *dsx* and *fru* encode transcription factors (TFs) that exert regulatory function by influencing downstream gene expression programs [17,18]. *dsx* transcripts undergo sex-specific alternative splicing that give rise to sex-specific Dsx isoforms: DsxF and DsxM in females and males, respectively [19]. In contrast, the sex-specific alternative splicing of *fru* transcripts is predicted to only produce functional FruM in males [20,21]. The presence of an additional female-specific exon in female *fru* transcripts introduces a premature stop codon predicted to yield a truncated Fru protein [20,21].

Prior work investigating FruM in *Aedes aegypti* (*Ae. aegypti*) mosquitoes found that *fruM* mutant males develop female-like host-seeking behavior, suggesting that FruM inhibits female behaviors in males [21]. Mutant males also exhibit mating failures and do not inseminate females, though the underlying causes remain unknown [21].

Here, we found that *fruM* mutant males showed altered flight trajectories compared to control males and completely lacked male phonotaxis behaviors, preventing mutant males from successful mating. Anatomical profiling confirmed strong, broad expression of *fru* in male JO neurons, suggesting *fru* could influence male hearing function, which underlies male hearing behaviors. Functionally, the loss of FruM dramatically altered several key hearing parameters in mutants compared to control males, including the complete abolition of male ear-unique self-sustained oscillations (SOs) that reflect male amplification of female flight sounds at 1000s-fold above baseline, as well as a strong reduction in auditory neural responses to frequency-specific stimuli.

Our anatomical and molecular profiling suggests that the altered hearing function of *fruM* males could arise from both changes in the efferent control of mutant ears and intrinsic changes in the FruM-dependent hearing cellular machineries. Through motif enrichment and protein-DNA interaction analyses, we identified a putative FruM-binding DNA motif that allowed for identification of genes to which FruM may directly bind. We confirmed FruM binding to a female-biased hearing-related gene whose enhancer region contains two putative FruM-binding DNA motifs. Our work lays the groundwork for future investigation of FruM-dependent mechanisms that define sexual dimorphisms in mosquito peripheral hearing.

We conclude that FruM is the master regulator of male hearing behaviors. FruM directs multilayered transcriptional networks to influence male-specific hearing function and behaviors, with hearing representing the primary sensory system involved in mosquito mating. FruM’s role in promoting male phonotaxis may not be restricted to downstream, central processing of acoustic stimuli. Instead, FruM may directly influence male phonotaxis by modulating the performance of the male peripheral auditory system, where active auditory mechanotransduction and sound detection first occur. We propose the mosquito peripheral auditory system as a new model for sex determination research and highlight *fru* as a novel promising target for mosquito control.

## Results

### FruM mutation abolishes male central flight pattern and phonotaxis

Male *Ae. aegypti* mosquitoes swarm at dusk to find mates [5,22]. Mating is initiated when males detect female flight sounds and rapidly approach females for mating, a hearing behavior known as “phonotaxis” (Fig 1A) [3]. Females neither swarm nor show phonotaxis [3].

**Fig 1:**
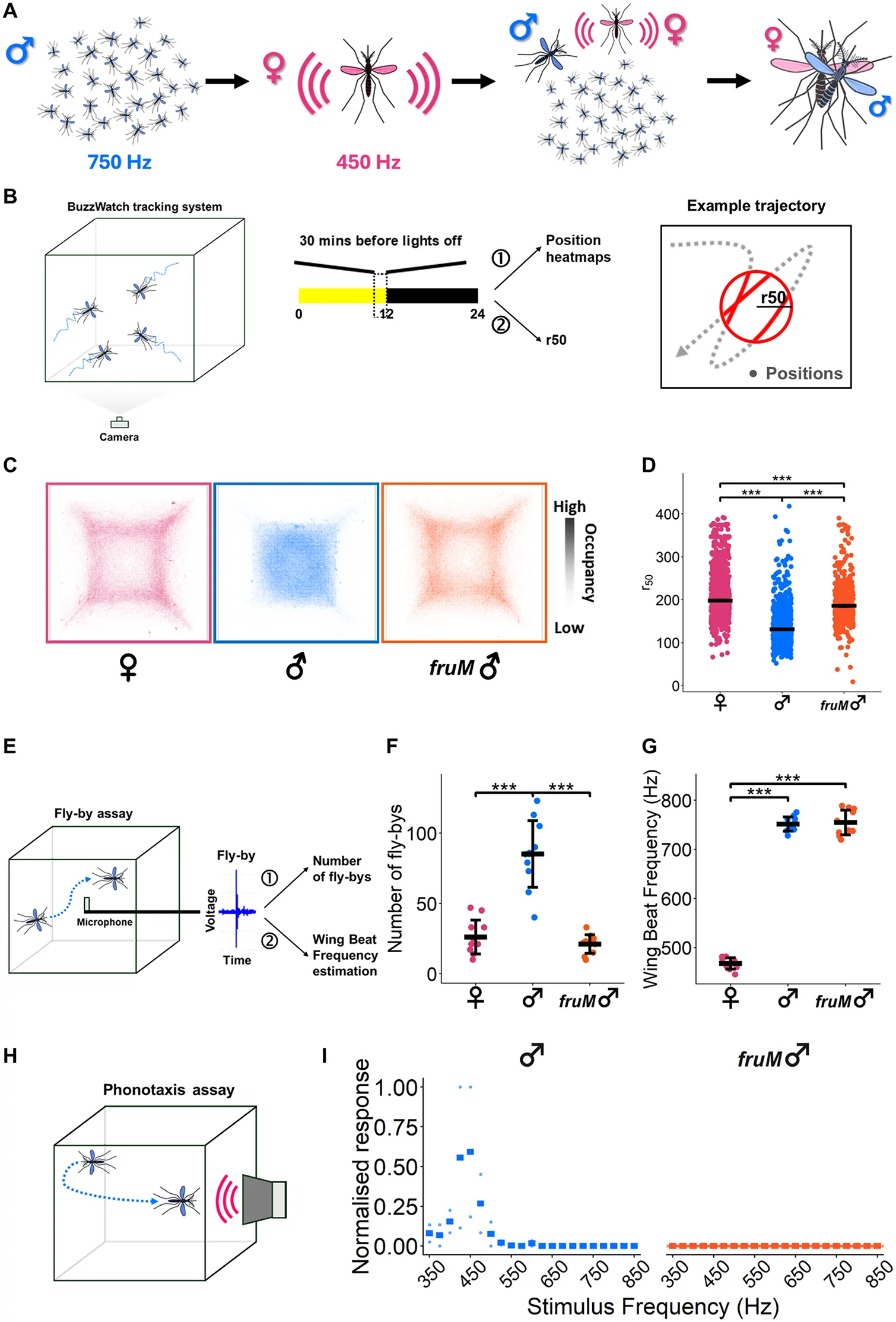
Loss of FruM abolishes male central flight pattern and phonotaxis. **(A)** Schematic of male aggregation and male attraction to female flight sounds. **(B)** Schematic illustration of BuzzWatch flight tracking system [24] and analysis paradigms. Mosquito flight trajectories during the 30-min period before lights-off were analyzed to extract the trajectory positions and *r*_50_ values (for each trajectory, the minimum distance from cage center in pixels that contained 50% of total trajectory positions). **(C)** Combined heatmaps of trajectory positions of control female (left), control male (center) and *fruM* male (right) mosquitoes between ZT11.5–ZT12. Color bar intensity from dark to light reflects high to low position occupancy in the cage. Three cages per genotype, 30 mosquitoes per cage. **(D)** r_50_ quantification for each individual trajectory obtained during ZT11.5–ZT12. Individual points represent individual trajectories. Solid lines represent medians and standard errors. See S2 Table. *** *p* < 0.001, pairwise Wilcoxon tests with BH correction. Sample sizes: 785 control female trajectories; 860 control male trajectories; 489 *fruM* male trajectories. **(E)** Wing Beat Frequency (WBF) and fly-by assay diagram. **(F)** Number of fly-bys per cage for control female, control male and *fruM* male groups. Individual points represent total number of fly-bys for individual cages. Solid lines represent means and standard deviations. See Tables 1 and S3. *** *p* < 0.001, pairwise *t*-tests with BH correction. Sample sizes: 11 control female cages; 11 control male cages; 11 *fruM* male cages. **(G)** WBFs of control females, control males and *fruM* males. Individual points represent median WBF estimates for one cage. Solid lines represent means and standard deviations. See Tables 1 and S4. *** *p* < 0.001, pairwise *t*-tests with BH correction. Sample sizes: 11 control females; 11 control males; 11 *fruM* males. **(H)** Phonotaxis assay diagram. **(I)** Normalized frequency response profiles of control and *fruM* males to a range of pure tones (350–850 Hz in 25 Hz bins). Bars represent median normalized response per frequency across all repeats. Individual points represent individual repeat normalized frequency responses. Sample sizes: 2 control male cages; 5 *fruM* male cages. 30 mosquitoes per cage. The data underlying this figure can be found at https://doi.org/10.17632/dtscz72px6.1.

**Table 1:** Parameters associated with flight and vibrometry for all groups tested. All values provided as means  ±  standard deviations. Numbers in brackets refer to sample sizes for each experiment.

Group	Fly-by number	Wing Beat Frequency (Hz)	Peak mechanical tuning frequency – White Noise (Hz)	Peak mechanical tuning frequency – Sedated (Hz)
Control female	26.09 ± 12.07 (*n* = 11)	467.83 ± 11.50 (*n* = 11)	245.18 ± 14.63 (*n* = 15)	234.37 ± 22.25 (*n* = 15)
Control male	85.18 ± 23.64 (*n* = 11)	751.53 ± 14.35 (*n* = 11)	397.92 ± 20.38 (*n* = 19)	324.40 ± 27.67 (*n* = 19)
*fruM* male	21.09 ± 6.64 (*n* = 11)	754.90 ± 25.05 (*n* = 11)	488.50 ± 61.40 (*n* = 12)	330.53 ± 29.79 (*n* = 12)

*fruM* mutant males exhibit mating failures, with unclear underlying causes [21]. To investigate this, we tested published heteroallelic *fruM* null mutant (*fruitless*^*∆M*^*/fruitless*^*∆M-tdTomato*^) males (hereafter denoted “*fruM* males”) [21].

Since locomotion and flight are essential for mating, we first tested the baseline locomotor activity level of *fruM* males in a 12:12 Light:Dark (LD) cycle (S1A Fig). *fruM* males exhibited bimodal peaks in locomotor activity at dawn (Zeitgeber Time (ZT)0) and dusk (ZT12), like control males and females (S1B Fig). However, *fruM* males showed a significant reduction in locomotor activity at dusk (ZT11–ZT12) compared to both control males and control females (pairwise Wilcoxon test with Benjamini–Hochberg (BH) correction; *p* = 2.30 × 10^−5^ and *p* = 2.15 × 10^−3^; S1 Table; S1B and S1C Figs). This matches the reported phenotype of *fruM* male mutants in *Drosophila melanogaster* (*D. melanogaster*) [23].

Male aggregation at dusk is crucial for mating [5]. The reduction in *fruM* male locomotor activity at dusk may reflect changes in this behavior. To investigate aggregation, we recorded cages of mosquitoes in the last 30 min before lights off to examine their flight patterns. We found that whilst control males tended to fly near the cage center, control females and *fruM* males typically flew along the cage sides (S1 Video).

We confirmed our observations using the BuzzWatch tracking system [24] (Figs 1B and S2A; S2 Video). Heatmaps based on recordings of cages of 30 mosquitoes at dusk (ZT11.5–ZT12) indicate that while the flight positions of control males were concentrated near the center of the cage, *fruM* males and control females were distributed along the edges (Figs 1C and S2B), with qualitative examples of individual trajectories supporting these differences (S3A–S3C, S4A–S4C and S5A–S5C Figs). To quantify these differences, we calculated the distributions of trajectory positions in the cage by computing the r_50_ value for each trajectory, defined for each trajectory as the radius from the cage center encompassing 50% of all trajectory positions. We found that control males had significantly lower r_50_ values at dusk than both control females and *fruM* males (pairwise Wilcoxon test with BH correction; *p* = 0 and 1.61 × 10^−94^, respectively; Fig 1D; S2 Table).

We next checked dawn (ZT0–ZT0.5), midday (ZT5.75–ZT6.25) and dusk to investigate if r_50_ values changed across the day. By calculating weighted averages for *r*_1_–*r*_100_ values (i.e., radii covering 1%–100% of all trajectory positions), we found that male flight positions appeared closer to the cage center at both dawn and dusk than control females and *fruM* males (S6A and S6B Figs). Our results indicate that FruM is essential in shaping the timely-regulated central flight pattern of males during dawn and dusk.

Male phonotaxis has been reported to depend on female and male Wing Beat Frequencies (WBFs), with male mosquitoes using both flight sounds to identify conspecific females [5,25]. We next placed a microphone into the center of a cage housing circadian-entrained, single-sex, free-flying mosquitoes to record and extract: (1) the number of flyby events past the microphone, an indicator of male aggregation at the cage center, and (2) mosquito WBFs, during dusk (Fig 1E). We found that the number of flybys for *fruM* males was significantly lower than control males but similar to control females (pairwise *t*-tests with BH correction; *p* = 3.58 × 10^−6^ and *p* > 0.05; Fig 1F; Tables 1 and S3). We found no significant difference in WBF between control and *fruM* males (pairwise *t*-tests with BH correction; *p* > 0.05), though control females had significantly lower WBFs than both male groups (*p* = 2.02 × 10^−21^ and *p* = 8.34 × 10^−15^; Fig 1G; Tables 1 and S4). We conclude that FruM controls male central flight pattern at dawn and dusk, but does not influence male WBFs.

Since male attraction to female flight sounds is a prerequisite for courtship, *fruM* male mating failure could be due to primary defects in phonotaxis [21]. Using a phonotaxis assay to test male responses to a broad range of pure tones encompassing and extending beyond the female WBF range (Fig 1H), we found that *fruM* males failed to show attraction to all tones tested, while control and heterozygous *fruM* (*fru*^*∆M*^/+) males were strongly attracted to specific tones (Figs 1I and S7A).

Furthermore, we found that another aspect of sound response behavior in males, sound-induced abdominal bending behavior [26] was also abolished in *fruM* males; when directly presented with various tones (including 400, 450, 500 and 550 Hz to cover the range of estimated control female WBFs, as well as 470 Hz to match the mean estimated female WBF), only control males showed sound-induced abdominal bending behavior (S3–S7 Videos).

We conclude that FruM is the master regulator of male *Ae. aegypti* hearing behaviors which play crucial roles in reproduction, specifically phonotaxis and sound-induced abdominal bending behavior.

### JO neurons are *fru* positive and FruM mutation alters male hearing function

Male peripheral hearing function underlies male hearing behaviors, including phonotaxis [26]. We therefore hypothesized that the complete abolition of male hearing behaviors in *fruM* males could be attributable to defects in mutant male peripheral hearing function.

We first examined *fru* expression in published wild-type male and female *Ae. aegypti* snRNA-seq and pedicel RNAseq databases [27,28], and found strong, broad *fru* expression in the putative JO neuron cluster (i.e., auditory neuron cluster) (Fig 2A). We further confirmed that *fru* transcripts in the pedicel undergo sex-specific alternative splicing (Figs 2B and S8A) [21,28].

**Fig 2:**
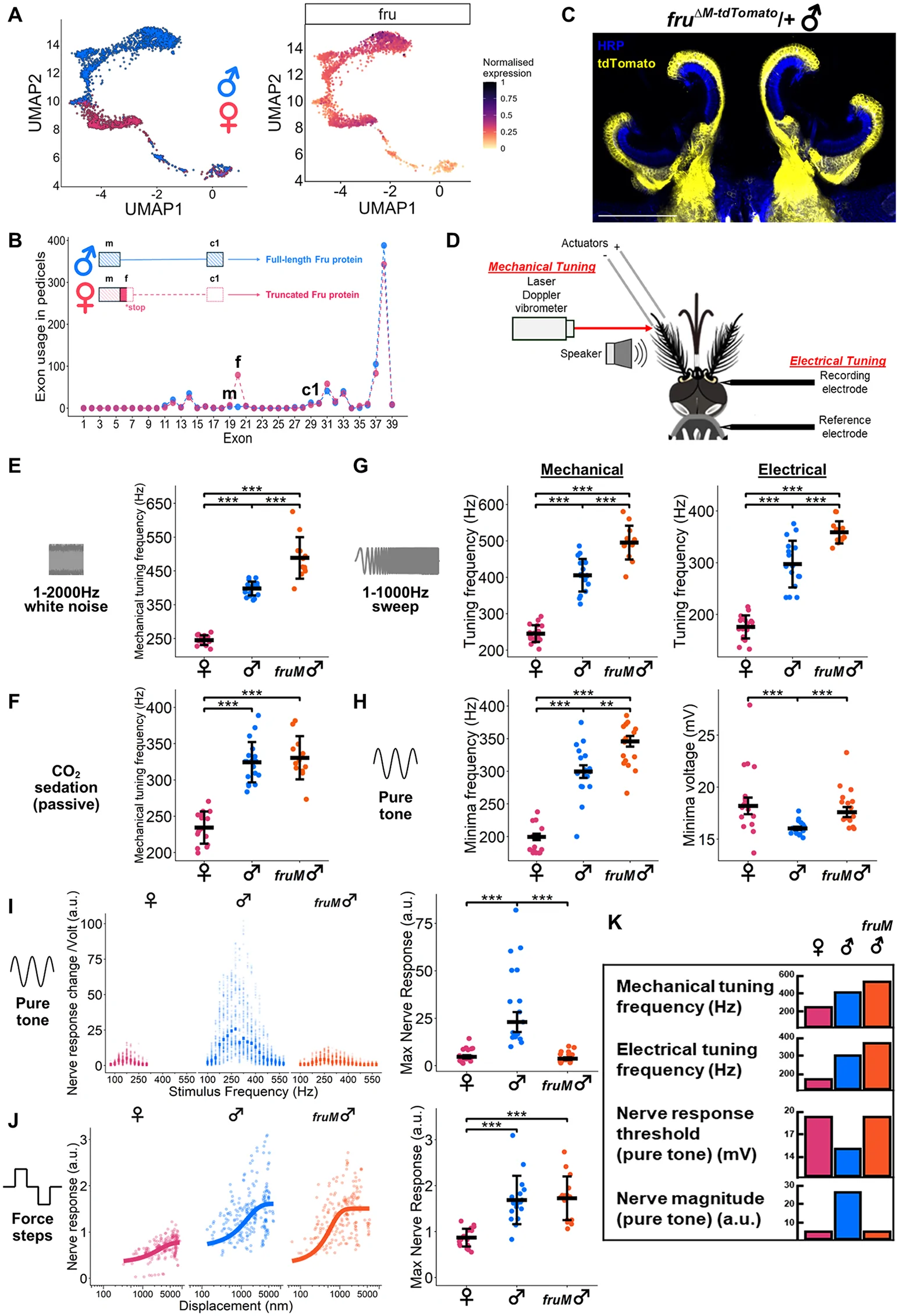
Loss of FruM alters male hearing function. **(A)** UMAP of putative male and female JO neurons of *Ae. aegypti* colored by sex (left) and normalized expression of *fru* (right). Data from [27]. Putative JO neurons were identified based on *inactive* (AAEL020482) expression. **(B)** Sex-specific alternative splicing of *fru* transcripts from reanalysis of published adult male and female *Ae. aegypti* pedicel transcriptome data [28]. **(C)** Expression of *fru* in *fru*^*ΔM-tdTomato*^/+ male JO. Scale bar, 100 μm. Maximum intensity projection of JOs stained with anti-tdTomato (yellow) and anti-HRP (blue). See S8B Fig for split-channel images. **(D)** Combined laser Doppler vibrometry and electrophysiology assay schematic. When measuring mechanical tuning alone, only laser Doppler vibrometer was used for recordings and stimulus was provided *via* a speaker. When simultaneously measuring mechanical and electrical tuning, stimulus was provided *via* actuation and electrodes were inserted into the base of the pedicel to measure sound-evoked compound action potentials in the antennal nerve. **(E)** Active, white noise stimulated mechanical tuning frequencies of control females, control males and *fruM* males. Individual points represent measurements from individual mosquitoes. Solid lines represent means and standard deviations. See Tables 1 and S5. *** *p* < 0.001, pairwise *t*-tests with BH correction. Sample sizes: 15 control females; 19 control males; 12 *fruM* males. **(F)** Passive, unstimulated mechanical tuning frequencies of control females, control males and *fruM* males. Individual points represent measurements from individual mosquitoes. Solid lines represent means and standard deviations. See Tables 1 and S6. *** *p* < 0.001, pairwise *t*-tests with BH correction. Sample sizes: 15 control females; 19 control males; 12 *fruM* males. **(G)** Active, sweep stimulated mechanical (left) and electrical (right) tuning frequencies of control females, control males and *fruM* males. Individual points represent measurements from individual mosquitoes. Solid lines represent means and standard deviations. See Tables 2, S8 and S9. *** *p* < 0.001, pairwise *t*-tests with BH correction. Sample sizes: 19 control females; 17 control males; 13 *fruM* males. **(H)** Minima electrical tuning frequencies (left) and minima voltage threshold responses (right) from threshold pure tone stimulation experiments for control females, control males and *fruM* males. Individual points represent electrical tuning frequency estimates for individuals. Solid lines represent medians and standard error. See Tables 2, S10 and S11. ** *p* < 0.01, *** *p* < 0.001, pairwise Wilcoxon tests with BH correction. Sample sizes: 17 control females; 17 control males; 16 *fruM* males. **(I)** Nerve phasic responses of control females, control males and *fruM* males from pure tone stimulation experiments. Individual points represent individual responses (nerve response magnitude normalized to stimulus voltage) to pure tones of the largest voltage intensity. Bars represent median responses to pure tones (left). Quantification of the maximum nerve phasic response for each individual tested (right). Solid lines represent medians and standard errors. See S12 Table. *** *p* < 0.001, pairwise Wilcoxon tests with BH correction. Sample sizes: 17 control females; 17 control males; 16 *fruM* males. **(J)** Nerve tonic responses of control females, control males and *fruM* males from force-step stimulation experiments. Individual points represent individual nerve responses to maximum flagellar displacements within an individual. Solid lines represent sigmoid fits (left). Quantification of the maximum nerve tonic response for each individual tested (right). Solid lines represent means and standard deviations. See S13 Table. *** *p* < 0.001, pairwise *t*-tests with BH correction. Sample sizes: 17 control females; 17 control males; 15 *fruM* males. **(K)** Summary diagram of hearing parame*t*ers across genotypes. The data underlying this figure can be found at https://doi.org/10.17632/dtscz72px6.1.

One of the two *fru* heteroallelic lines carries a tdTomato marker expressed under the control of the endogenous *fru* promoter, enabling visualization of *fru*-expressing neurons [21]. We stained *fruitless*^*∆M-tdTomato*^*/*+ male JOs with anti-tdTomato and found extensive *fru* expression, consistent with snRNA-seq and pedicel RNAseq observations (Figs 2C and S8B). Brain immunostaining of *fruitless*^*∆M-tdTomato*^*/*+ males and females showed strong tdTomato expression in the antennal mechanosensory and motor center (AMMC) to which JO neuron axons project [28,29] (S9A and S9B Figs), confirming that JO neurons are *fru* positive.

FruM expression in male JOs may shape male-unique hearing function supporting male hearing behaviors. Therefore, we next elucidated FruM’s influence on male peripheral hearing function.

Males use active hearing to detect female flight tones [30–32]. Mechanical deflections of the flagellum stretch-activate mechanotransducers in JO neurons to elicit action potentials that relay auditory information to the brain [33]. Mosquito hearing is a bidirectional process: in addition to mechanotransducing sound, JO neurons actively inject energy to set flagellae in motion even in the absence of sound [33]. This energy-dependent mechanical feedback amplification defines their baseline frequency sensitivity and selectivity for behaviorally relevant sounds [30–32].

The free-fluctuating male flagellum actively vibrates at frequencies that encompass the range of female WBFs [33]. Male ears actively amplify female flight sounds, which increases male hearing sensitivity and prepares males for when females enter their audible zones [25]. Frequency ranges of male and female hearing amplification appear different; male hearing amplification supports male phonotaxis, but the biological significance of female amplification is unclear [28].

We first asked if FruM is involved in establishing male ear tuning frequency in response to sound. The frequency tuning properties of mosquito flagellar ears are set by active JO neuron motility [33]. Using a laser Doppler vibrometer, we focused a laser beam at the tip of the mosquito flagellum and measured flagellar vibration velocity over time (Fig 2D). After transforming this data from time to frequency domains, we fit a force-damped harmonic oscillator function to the frequency domain data and extracted the peak mechanical frequency (denoted the mechanical tuning frequency).

We found significant differences between male and female stimulated mechanical tuning frequencies in response to broadband white noise (pairwise *t*-tests with BH correction; *p* = 3.36 × 10^−22^; Fig 2E; Tables 1 and S5). *fruM* males showed a significantly higher stimulated mechanical tuning frequency than control males (pairwise *t*-tests with BH correction; *p* = 2.99 × 10^−4^; Fig 2E; Tables 1 and S5).

Active energy injection by JO neurons can be reversibly suppressed *via* CO_2_ sedation, enabling measurement of flagellar passive movements in response to background thermal bombardment. Measurements in the passive state can therefore reveal structural and morphological differences of the flagellar ears between sexes which could not be uncovered with awake, active state recordings [33].

Consistent with the lack of apparent anatomical differences between control and *fruM* male ears [21], passive ear mechanical tuning frequencies of male groups did not significantly differ from each other but were significantly different from control females (pairwise *t*-tests with BH correction; *p* > 0.05 for comparisons between males; *p* = 1.98 × 10^−11^ and *p* = 1.66 × 10^−8^; Fig 2F; Tables 1 and S6). We confirmed that the difference in frequency tuning between stimulated active and unstimulated passive states was significantly greater for *fruM* males than control males (pairwise *t*-tests with BH correction; *p* = 9.96 × 10^−4^; S10A Fig; S7 Table). Our passive state measurements confirmed that active state mechanical tuning frequency differences between control and *fruM* males are indeed determined by active JO neuron properties.

Male mosquito mechanical tuning frequency is level-dependent, with male mechanical tuning frequency decreasing as stimulus amplitude increases [33]. Regardless of the presence or absence of sound stimulation, we found that control male ear mechanical tuning frequency ranges covered the entire female WBF range, suggesting that control male ears are intrinsically tuned to and actively amplifying female flight sounds regardless of acoustic conditions (Figs 2E, S10B and S10C). On the other hand, we observed minimal overlap between *fruM* male ear mechanical tuning frequencies and female WBFs, revealing defects in mutant male ears in amplifying female flight sounds (S10C Fig).

After profiling ear mechanical vibrational properties, we next investigated ear electrical properties *via* electrophysiology (Figs 2D and S11A). We simultaneously measured electrical responses from the antennal nerve [26], through which JO neurons relay signals to the brain, and ear mechanical vibrations. We started by quantifying responses to frequency sweep stimulation of 1–1,000 Hz and determined the ear electrical tuning frequency as the frequency eliciting the greatest compound action potential in the antennal nerve. We again found that *fruM* males had a significantly higher stimulated mechanical tuning frequency than control males (pairwise *t*-tests with BH correction; *p* = 1.51 × 10^−5^; Fig 2G; Tables 2 and S8). Consistent with the mechanical tuning frequency phenotype, *fruM* male ear electrical tuning frequency was also significantly higher than control males (pairwise *t*-tests with BH correction; *p* = 4.79 × 10^−5^; Fig 2G; Tables 2 and S9).

To quantify changes in antennal nerve response magnitude upon FruM mutation, we determined the smallest stimulus magnitude necessary to induce a detectable antennal nerve response by providing pure tone stimuli (25 Hz bin) at increasing stimulus intensities (S12A Fig) [26]. Once again, the electrical tuning frequency of *fruM* males was significantly higher than control males and females (pairwise Wilcoxon tests with BH correction; *p* = 4.60 × 10^−3^ and *p* = 5.14 × 10^−9^; Fig 2H; Tables 2 and S10). *fruM* males had a significantly higher electrical response threshold compared to control males but not control females (pairwise Wilcoxon tests with BH correction; *p* = 1.11 × 10^−4^ and *p* > 0.05; Fig 2H; Tables 2 and S11).

We observed that pure tone stimuli of identical intensities were able to elicit about 6-fold greater antennal nerve response in control males compared to *fruM* males and control females (pairwise Wilcoxon tests with BH correction; *p* = 9.00 × 10^−9^ for both comparisons; Figs 2I, S13A and S13B; S12 Table). However, static deflections of ears elicited similar magnitudes of antennal nerve response in *fruM* and control males (pairwise *t*-tests with BH correction; *p* > 0.05; Figs 2J and S14A; S13 Table).

The higher electrical response threshold and significantly reduced antennal nerve response of *fruM* male ears may reflect altered phasic responses of *fruM* male JO neurons to pure tone stimuli, potentially due to (feminized) alterations in mechanotransducer adaptation machinery [34,35]. The seemingly intact tonic response of *fruM* male JO neurons to static deflections suggests a similar number of functional mechanotransducer units between control and *fruM* male ears [33].

Collectively, sex-specific alternative splicing of *fru* in JO neurons suggests that FruM regulates male peripheral auditory processing by establishing the frequency sensitivity and selectivity of male ears to female flight sounds (Fig 2K).

### FruM influences localization of efferent presynaptic terminals in male JOs

We next characterized JO neuroanatomy *via* immunofluorescence, as sexual dimorphisms in hearing function are partly attributable to differences in JO neuroanatomy [33].

We observed significant differences in JO width between control males and females (reflecting the substantially greater number of JO neurons in male ears [29]), but not between control and *fruM* males, suggesting similar numbers of JO neurons in both male groups (pairwise *t*-tests with BH correction; *p* < 4.36 × 10^−10^ and *p* > 0.05, respectively; Fig 3A; S14 and S15 Tables). This matches our functional characterization of male ear passive systems, showing that structural and morphological properties of the flagellar ears remain similar between control males and *fruM* males (Fig 2F).

**Fig 3:**
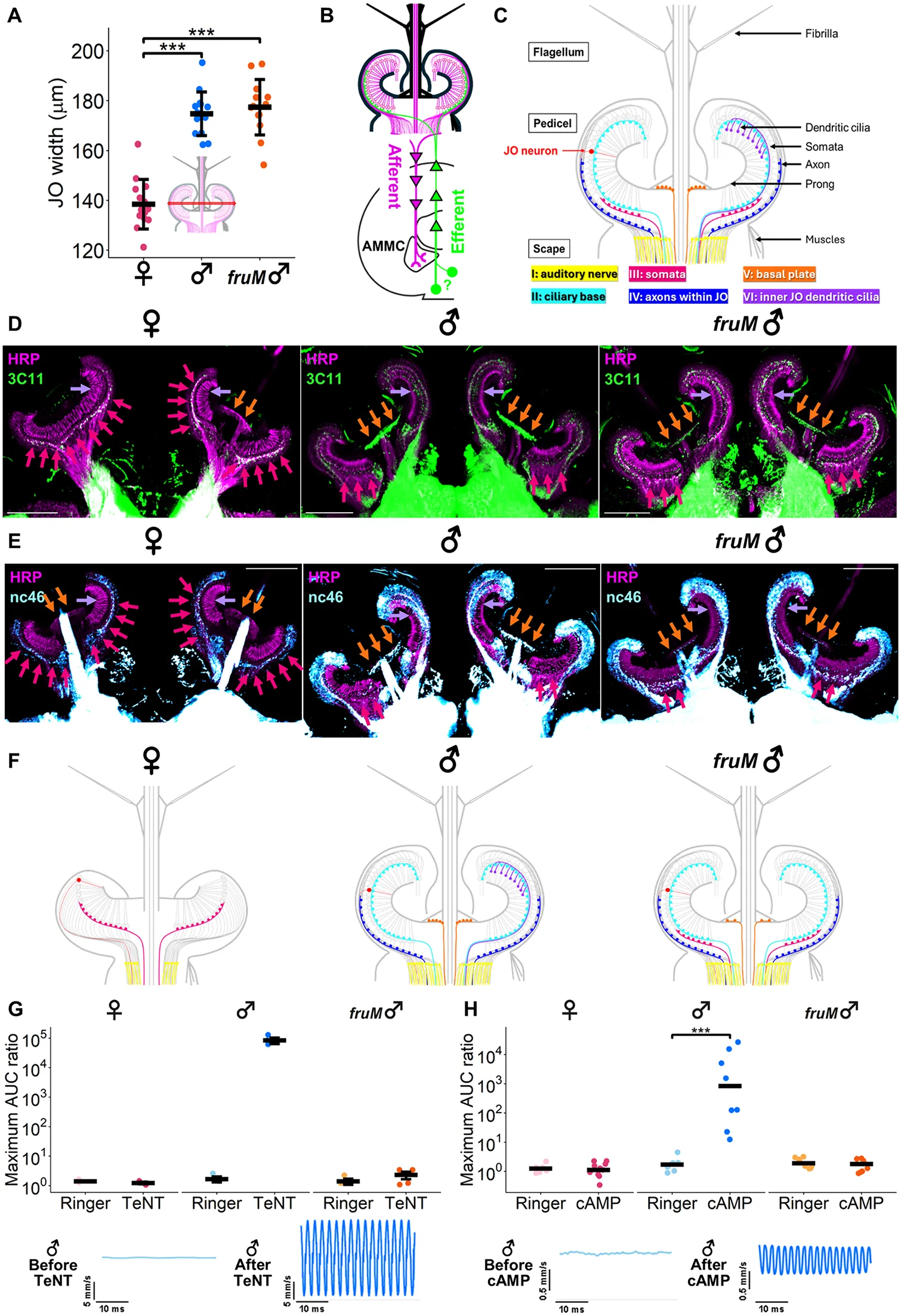
Loss of FruM alters localization of presynaptic terminals in male JOs and abolishes male SOs. **(A)** JO width of control females, control males, and *fruM* males. Individual points represent JO widths obtained from individual mosquito JOs. Solid lines represent means and standard deviations. See S14 and S15 Tables. *** *p* < 0.001; pairwise *t*-tests with BH correction. Sample sizes: 14 control females; 14 control males; 13 *fruM* males. **(B)** Schematic of afferent/efferent signaling in mosquito ear. Afferent signaling represents transduction and transmission of electrical signals from the JO to the brain, *via* synapsing of JO neuron axons onto downstream neurons in the antennal mechanosensory and motor center (AMMC) in the brain. Efferent signaling represents transmission of information from the brain to the JO *via* efferent neurons innervating distinct regions of the JOs. The origin of efferent neurons in the central nervous system remains unknown. **(C)** Schematic of the different classes of efferent presynaptic terminal sites in mosquito JO. Adapted from Loh and colleagues, 2023 [10]. Type VI terminal represents a new class first identified in this study. **(D)** Control female, control male and *fruM* male JO neuroanatomy visualized using immunofluorescence. Maximum intensity projections of JOs stained with anti-SYNORF1 (3C11, green) and anti-HRP (magenta). Scale bar, 100 μm. Red arrows = type III terminals (somata), orange arrows = type V terminals (basal plate), purple arrows = type VI terminals (inner JO dendritic cilia). See S15A Fig for split channel images. **(E)** Control female, control male and *fruM* male JO neuroanatomy visualized using immunofluorescence. Maximum intensity projections of JOs stained with anti-SAP47 (nc46, blue) and anti-HRP (magenta). Scale bar, 100 μm. Red arrows = type III terminals (somata), orange arrows = type V terminals (basal plate), purple arrows = type VI terminals (inner JO dendritic cilia). See S15B Fig for split channel images. **(F)** Summary schematic of presynaptic terminal locations across groups. Color coding of efferent terminal types follows Fig 3C. **(G)** Maximum ratio of AUC for vibrometry recordings taken before and after compound injection (Ringer or TeNT) for control females, control males and *fruM* males. Individual points represent data from individual mosquitoes. Solid lines represent medians. See S16 Table for maximum ratio of AUC following Ringer or TeNT injections (top). Representative control male flagellar vibrations before and after TeNT injection (bottom). Sample sizes for Ringer and TeNT injections: 3/3 control females; 3/3 control males; 3/4 *fruM* males. **(H)** Maximum ratio of AUC for vibrometry recordings taken before and after compound injection (Ringer or cAMP) for control females, control males and *fruM* males. Individual points represent data from individual mosquitoes. Solid lines represent medians. See S17 and S18 Tables for maximum ratio of AUC following Ringer or cAMP injections and *p* values. *** *p* < 0.001, Wilcoxon tests. (top). Representative control male flagellar vibrations before and after cAMP injection (bottom). Sample sizes for Ringer and cAMP injections: 7/10 control females; 8/8 control males; 8/8 *fruM* males. The data underlying this figure can be found at https://doi.org/10.17632/dtscz72px6.1.

Uniquely among insects, mosquito ears contain an auditory efferent system that can modulate JO neuron properties (Fig 3B) [9]. The efferent system is composed of descending neurons from the CNS that innervate different regions of the JO (Fig 3B) [10]. The localization of efferent presynaptic terminals within JO is sexually dimorphic [33]. We therefore tested if FruM influences male-specific localization of presynaptic terminals. To visualize presynaptic terminals, we stained JO sections with presynaptic marker antibodies 3C11 and nc46 [9].

Following previous classification guidelines (Fig 3C), we successfully identified all five types of presynaptic terminals in our JO sections [10]. We additionally observed presynaptic terminals located on the inner segments of JO dendritic cilia in control males, which we classified as type VI presynaptic terminals. By comparing between control males and females, we observed female-specific type III presynaptic terminals and male-specific type II, IV, V and VI terminals (Figs 3D, 3E, S15A and S15B). Type I terminals were observed in both control groups (Figs 3D, 3E, S15A and S15B).

We observed differences in *fruM* male JO presynaptic terminal localization compared to control males: (1) male-specific type V terminals appeared less abundant in *fruM* males, (2) male-specific type VI terminals were not detected in *fruM* males and (3) *fruM* males gained female-specific type III terminals (Figs 3D, 3E, S15A and S15B; red arrows: III (somata), orange arrows: V (basal plate), purple arrows: VI (inner JO dendritic cilia)). Using SynBot, we quantified numbers of Type III and Type VI punctae in the JO (S16A Fig). We found that control females and *fruM* males had more type III punctae than control males but lacked type VI punctae (Figs 3F and S16A).

Sexual dimorphisms in efferent innervation of JO neurons are associated with efferent modulation of hearing function [33]. The male efferent system controls the onset and cessation of male ear-unique self-sustained oscillations (SOs), which are large, monofrequent flagella vibrations reflecting excessive amplification of female flight sounds hundreds to thousands of times above baseline, a phenomenon associated with enhanced JO neuron motility [32,33]. The injection of tetanus toxin (TeNT) blocks efferent signaling by inhibiting the presynaptic release of neurotransmitters, triggering SO onset only in males [33]. Following TeNT injection, we found that only control males could show SOs, with *fruM* males not exhibiting any SOs (Figs 3G and S17A; S16 Table).

We next injected 8-Br-cAMP, which can induce SOs by direct activation of JO neurons [12]. Again, we found that cAMP injection was able to induce SOs only in control males (Fig 3H and S17 and S18 Tables), though injection increased the mechanical tuning frequency of all groups (S17B and S18A–S18F Figs; S17 and S19 Tables). This matches prior work indicating that exposure to cAMP increases mechanical and electrical tuning frequencies in both control male and female *Ae. aegypti*, but induces SOs only in control males [12].

Here, we found that FruM promotes proper localization and formation of male-specific efferent presynaptic terminals within male JOs and inhibits formation of female terminals. FruM could play a role in establishing male ear SO machinery or controlling the onset of male-unique SOs *via* direct modulation of JO neuron properties and/or indirect regulation of JO-innervating auditory efferent system properties.

### FruM inhibits female-biased gene expression and modulates male-biased gene expression

To elucidate the transcriptional regulatory roles of FruM in shaping male-specific hearing function properties, we submitted pedicels for RNA-sequencing. Principal Component Analysis (PCA) showed that PC1 (42% of variance) was attributable to genotype, indicating genotype-specific gene expression (Fig 4A).

**Fig 4:**
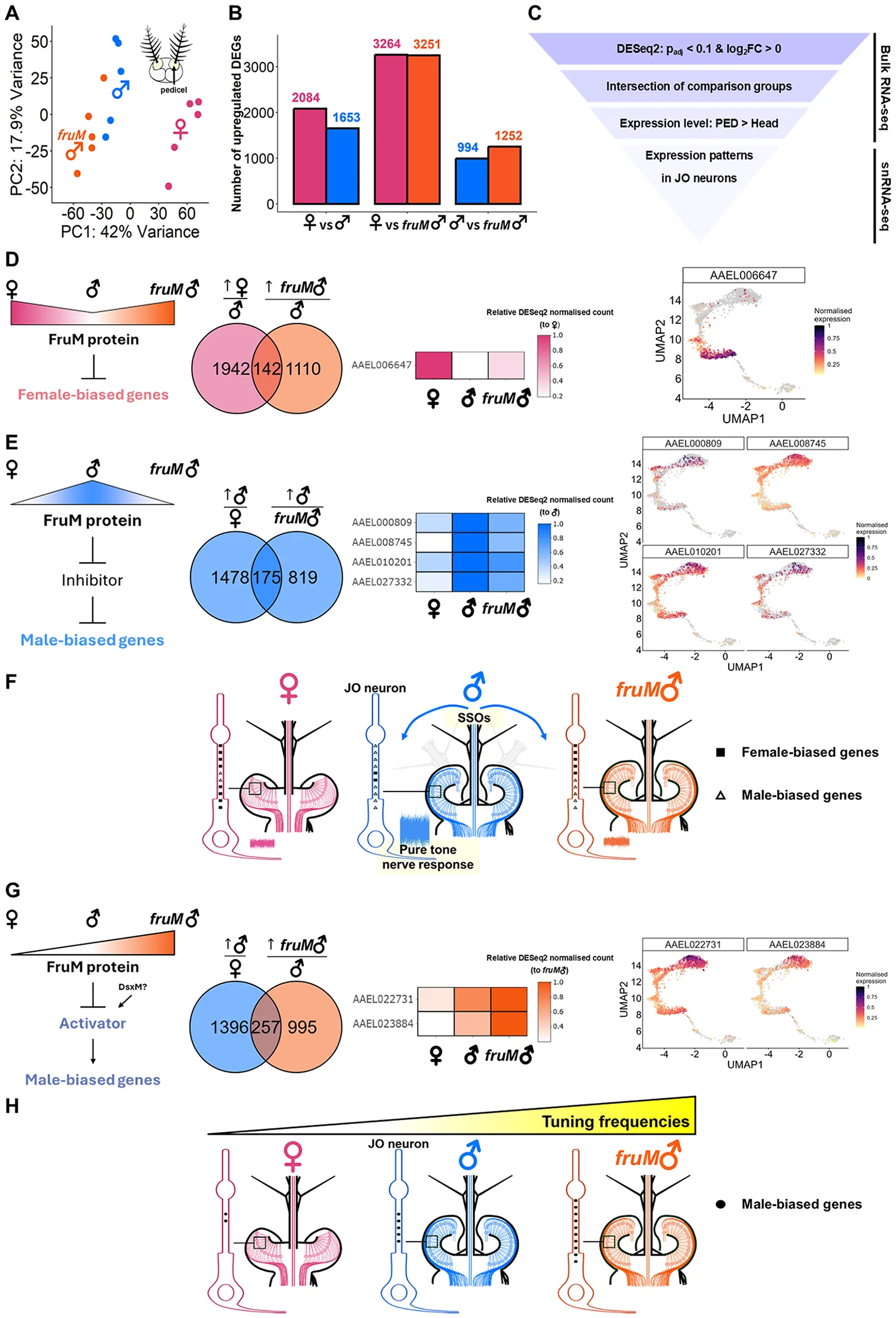
FruM-mediated transcriptional regulation. **(A)** Principal Component Analysis for control female, control male and *fruM* male pedicels. Pedicel dissection diagram with pedicels colored yellow (inset). **(B)** Bar chart of differentially expressed genes across comparison groups based on DESeq2 analysis. **(C)** RNAsequencing analysis pipeline. **(D)** Venn diagram of genes upregulated in control females and *fruM* males compared to control males (left); heatmap of target gene expression in pedicels of all groups (middle); UMAP showing expression pattern of AAEL006647 in JO neuron nuclei (right). See Fig 2A for the sex of JO neuron nuclei. Heatmap shows relative DESeq2 normalized counts (relative to females).**(E)** Venn diagram of genes upregulated in control males compared to control females and *fruM* males (left); heatmap of target gene expression in pedicels of all groups (middle); UMAP showing expression patterns of male-biased genes in JO neuron nuclei (right). See Fig 2A for the sex of JO neuron nuclei. Heatmap shows relative DESeq2 normalized counts (relative to males). **(F)** Schematic representation linking changes in target gene expression in s_142_ and s_175_ with changes in JO neuron function across groups. Loss of FruM results in upregulation of female-biased gene expression (represented by squares, shown in Fig 4D) and downregulation of male-biased gene expression (represented by triangles, shown in Fig 4E) compared to control males. These genes are highlighted due to their putative roles linked to ciliary motility, which powers active hearing in the antennal ears of insects. In *fruM* male ears, gain of female-biased gene expression may be linked to observed female-like antennal nerve responses to pure tone stimuli (phasic responses), while the loss of male-biased gene expression may be linked to the loss of male-specific SOs. **(G)** Venn diagram of genes upregulated in control males compared to control females and upregulated in *fruM* males compared to control males (left); heatmap of target gene expression in pedicels of all groups (middle); UMAP showing expression patterns of male-biased genes in JO neuron nuclei (right). See Fig 2A for the sex of JO neuron nuclei. Heatmap shows relative DESeq2 normalized counts (relative to *fruM* males). **(H)** Schematic representation linking changes in target gene expression in s_257_ with changes in JO neuron tuning frequencies across groups. Loss of FruM results in upregulation of male-biased gene expression (represented by circles, shown in Fig 4G) compared to control males. These genes are highlighted due to their putative roles linked to ciliary motility, which powers active hearing in the antennal ears of insects. In *fruM* male ears, the gain of male-biased gene expression compared to control males could be associated with the further increase in mechanical and electrical tuning frequencies. The data underlying this figure can be found at https://doi.org/10.17632/dtscz72px6.1.

Differential gene expression analysis between *fruM* and control males identified 1,252 genes significantly upregulated in *fruM* males compared to control males and 994 significantly downregulated genes (Fig 4B). Gene ontology (GO) enrichment analysis revealed that upregulated genes in *fruM* males had molecular functions related to histone modifications and microtubule motor activity, whilst downregulated genes in *fruM* males were enriched with functions related to cellular respiration and mitochondrial activity (S19A and S19B Figs).

To identify pedicel-specific genes potentially influenced by FruM (Fig 4C), we: (1) identified significantly differentially expressed genes (padj < 0.1 and log_2_FC > 0 (upregulated) or log_2_FC<0 (downregulated)) (S20–S23 Tables), (2) filtered for genes with higher expression in pedicels than heads in either sex using published head and pedicel RNA-seq databases [28] and (3) examined expression patterns in putative JO neurons using a published snRNA-seq database [27].

We started by searching for genes differentially expressed in the absence or presence of functional FruM. We identified 142 genes significantly upregulated in *fruM* males and control females compared to control males (s_142_) (Figs 4D and S20A). In s_142_, we found an important dynein gene, *dynein light chain 90F* (*Dlc90F*; AAEL006647), showing highly female-biased expression in JO neurons (log_2_FC[control females/control males] > 2.26, padj = 4.58 × 10^−83^) (Fig 4D). As *Dlc90F* was also upregulated in *fruM* males compared to control males (log_2_FC[*fruM* males/control males] > 0.89, padj = 1.06 × 10^−11^), FruM could be inhibiting the expression of this gene in control male JO neurons, consistent with the role of FruM as a transcriptional repressor in *D. melanogaster* [36]. Given that dynein-driven ciliary motility shapes active hearing properties of antennal ears [34,37,38], AAEL006647 could be involved in shaping female-specific hearing machineries, such as the mechanotransducer adaptation machinery potentially responsible for phasic responses of female JO neurons to pure tone stimulation, which we found to be similar in control females and *fruM* males compared to control males (Fig 4F).

We further identified 175 genes significantly upregulated in control males compared to *fruM* males and control females (s_175_) (Figs 4E and S20B). In s_175_, we found four genes showing male-biased expression patterns in JO neurons (Fig 4E). These genes encode components of the ciliary motility apparatus that powers JO neuron motility, such as dynein intermediate chains, dynein docking and regulatory subunits [34,37,38]. These genes could therefore shape male-unique acoustic physiology, such as male-specific SOs (hearing amplification) as well as male-enhanced mechanical and electrical sensitivities to sounds (Fig 4F).

Given that we observed an increase in mechanical and electrical tuning frequencies from control females to control males to *fruM* males, we further searched for genes that showed changes in expression following the same trend (Fig 4G). We identified 257 dose-dependent genes (s_257_) (Figs 4G and S20C), including two dynein heavy chains (*Dhc;* AAEL023884 and *Dhc93AB*; AAEL022731). Functional research in *D. melanogaster* has demonstrated that loss of *Dhc93AB* results in abolished mechanical amplification and lack of sound-evoked compound action potentials in the antennal nerve [37]. These genes could therefore be involved in the genotype-dependent increase in ear tuning frequencies (Fig 4H).

Collectively, FruM could act as a primary transcriptional repressor to inhibit female-biased gene expression in males, and by indirect regulation (such as exerting transcriptional control over intermediate TFs) could also promote or modulate the expression of male-biased genes (Figs 4D, 4E and 4G).

### Motif enrichment analysis identified putative FruM-binding DNA motifs exhibiting interactions with FruM protein’s DNA-binding domain

FruM proteins are TFs containing a BTB domain on their N-termini for protein-protein interactions and one of three C_2_H_2_ zinc-finger domains on their C termini for sequence-specific DNA binding [39–41]. Alternative splicing of *fru* transcripts at the 3′ end gives rise to different FruM protein isoforms (FruMA, FruMB or FruMC), depending on the zinc-finger domain being incorporated [40].

To identify putative FruM-binding genes to validate our hypothesis that FruM may be acting as a transcriptional repressor of genes in s_142_, we first focused on identifying putative FruM-binding DNA motifs. We conducted motif enrichment analysis on 1kbp enhancer sequences upstream of translational start sites (TSS) of genes in s_142_, s_175_ and s_257_ (Fig 5A). The identified motifs showed minimal overlap between gene groups (Fig 5A). We focused on motifs identified in s_142_, as this gene group was predicted to be under the direct transcriptional inhibition of FruM. We conducted automated motif clustering based on sequence similarity and found a cluster composed of three members whose consensus motif showed 7/10 base pair identity with the FruMB-binding motif in *D. melanogaster* (Figs 5B and 5C) [20].

**Fig 5:**
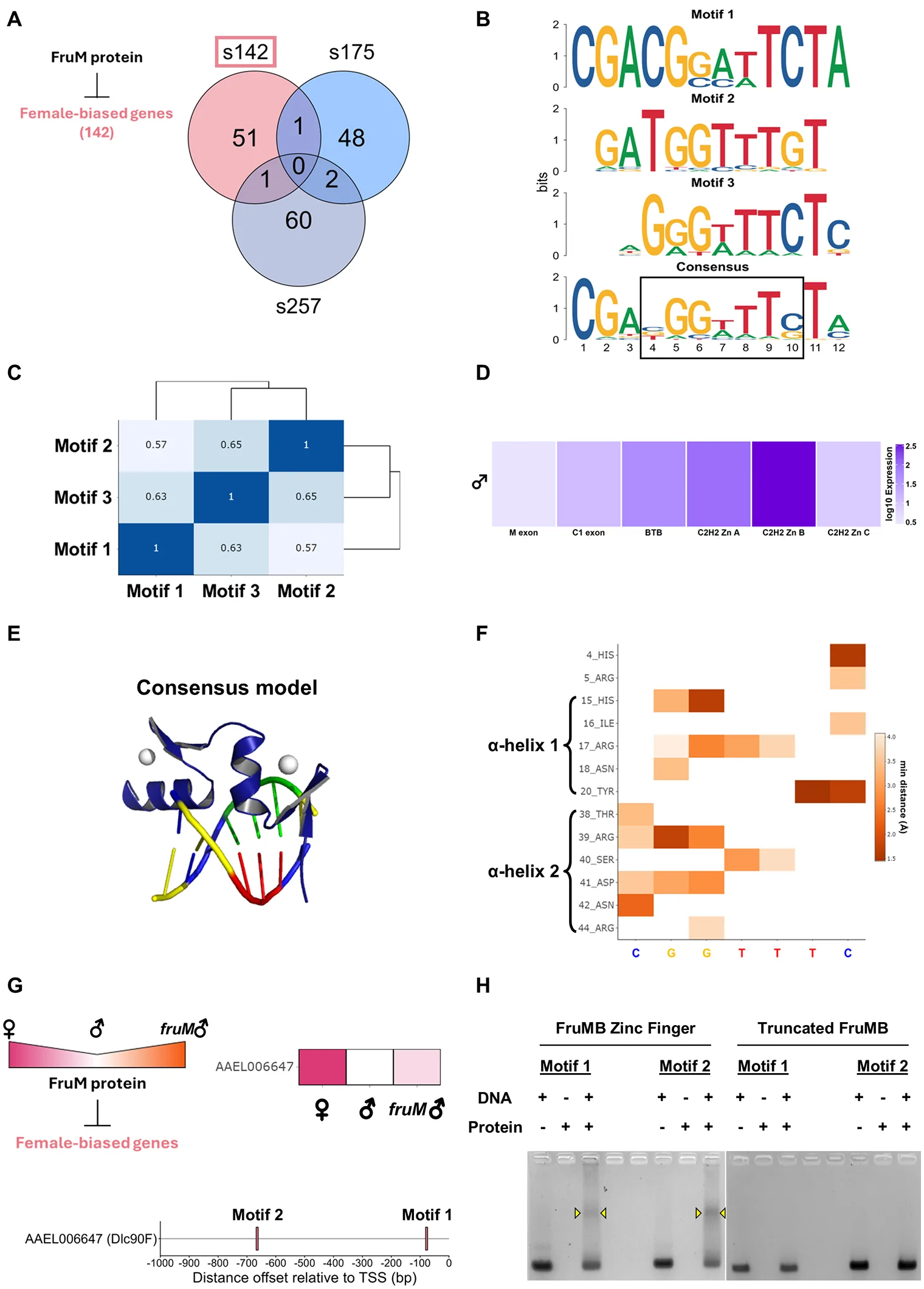
Identification of putative FruM-binding motifs. **(A)** Venn diagram showing motifs enriched in s_142_, s_175_ and s_257_. **(B)** Putative FruM-binding motif cluster identified based on automated motif clustering. Boxed nucleotide positions in the consensus motif are nucleotides that consistently showed interactions with Zn-B domain of FruM across all three members of the motif cluster (S21A Fig). **(C)** Pearson Correlation coefficients between members of FruM-binding motif cluster. **(D)** Heatmap of *fru* exon usage counts in male pedicels, from reanalysis of published wild-type adult male and female *Ae. aegypti* pedicel transcriptomes [28]. C_2_H_2_ Zn-A, Zn-B and Zn-C respectively refer to exons 37–39 of *fru* shown in Fig 2B. **(E)** AlphaFold3 model of Zn-B of FruM binding to consensus DNA motif. Two spheres represent two zinc-ions. **(F)** Minimal distance interactions between indicated amino acids of native Zn-B of FruM and nucleotide positions of consensus DNA motif. Only interactions with minimal distance <4.5 Å were plotted. Amino acid positions 15–20 form part of the first alpha helix of Zn-B and amino acid positions 38–44 form part of the second alpha helix. **(G)** Identification of putative FruM-binding DNA motifs in the 1 kbp enhancer region upstream of translational start site (TSS) of AAEL006647 (*Dlc90F*), with heatmap of AAEL006647 expression in pedicels of all groups. **(H)** EMSA data showing protein-DNA interactions between zinc-finger domain (Zn-B) of FruM (left) or truncated FruM without zinc-finger domain (right) and the two 100 bp enhancer fragments of AAEL006647 which each bear a centered putative FruM-binding DNA motif (see Fig 5G). Interactions represented as band shifts were observed only between Zn-B domain and the two 100 bp enhancer fragments. See S1 Raw Images for whole gel images. The data underlying this figure can be found in S1 Raw Images and at https://doi.org/10.17632/dtscz72px6.1.

From our bulk pedicel RNA-seq data, we found that in control males, the *fru* exon that encodes the type B C_2_H_2_ zinc-finger domain (hereafter referred to as Zn-B domain) also showed the highest expression among all three zinc-finger coding exons. FruMB could thus be the predominant FruM isoform in male JOs (Fig 5D).

We next used AlphaFold3 to predict interactions between the Zn-B of FruM and candidate motif members (S21A Fig) [42,43]. We performed pairwise distance analysis and, across three models, consistently identified seven core nucleotide positions with <4.5 Å interactions with the amino acid residues of Zn-B, which we referred to as the FruM-binding consensus DNA motif (S21A Fig). To examine the specificity of these interactions, we performed single-base transversion (A ↔ C or T ↔ G) scanning across the seven positions and identified three important positions (positions 2, 3 and 5) mediating these interactions, with dissociation of interactions observed upon mutation (Figs 5E and S21B).

We found that among the 13 amino acids of Zn-B that showed interactions with the consensus DNA motif, 9 had interactions with minimal distance <3.5 Å (Fig 5F). Four were part of the first alpha helix of Zn-B and five belonged to the second alpha helix, suggesting the importance of the zinc-finger alpha helices in mediating sequence-specific recognition of consensus DNA motif (Fig 5F) [44,45]. We introduced single-amino acid proline substitutions for each of these amino acids to break the alpha helix structure and identified three amino acids to be essential for N and C termini interactions with the consensus DNA motif (S21C Fig).

Our *in silico* results indicate that the C_2_H_2_ zinc-finger domain of FruMB exhibits sequence-specific binding with our putative FruM-binding consensus DNA motif.

### Putative FruM-binding consensus DNA motif enriched in enhancer sequences of genes predicted to be under direct FruM transcriptional inhibition

To help validate our *in silico* findings, we scanned for putative FruM-binding sites in the enhancer region of genes in s_142_ (genes potentially under the direct transcriptional inhibition of FruM) and the non-differentially expressed gene (non-DEG) group (non-differentially expressed between *fruM* and control males) using the identified FruM-binding consensus DNA motif. We applied the strict criterion that positions 2, 3 and 5 of identified target sites should be identical to the consensus motif (Figs 5B and S21B; S24 and S25 Tables). We identified significantly more genes in s_142_ (55.6%) containing at least one consensus motif in their enhancer region compared to the non-DEG group (40.9%) (Chi-squared test; *p* < 0.05; S21D Fig).

Among the genes in s_142_ that contain putative FruM-binding sites, we found AAEL006647 (*Dlc90F*), a putative FruM-inhibited female-biased hearing gene, with two FruM-binding sites within its 1kbp upstream enhancer region (Fig 5G). To examine if FruM could directly bind to these sites, we performed Electrophoretic Mobility Shift Assay (EMSA) to determine interactions between the zinc-finger domain (Zn-B) of FruM and the two 100 bp DNA sequences derived from the AAEL006647 enhancer region, each containing a centered FruM-binding DNA motif with native flanking sequences. We observed that the Zn-B of FruM could bind to both enhancer sites, evidenced by band shifts, with these interactions lost upon using truncated FruM protein lacking the Zn-B domain (Fig 5H), supporting the hypothesis that FruM may regulate expression of *Dlc90F* through direct binding.

## Discussion

Strong sexual dimorphisms in mosquito reproductive behaviors have been documented for almost a century [3,4]. Males form mating swarms and find females *via* attraction to female flight sounds. Females do not display these behaviors, but instead seek hosts, blood-feed and lay eggs [3,5–7]. However, the molecular underpinnings of these behavioral dimorphisms remain unclear. Here, we show that *fru* is the master regulator of male reproductive behaviors. The loss of FruM completely abolishes male-specific central flight pattern and phonotaxis, resulting in mating failures of *fruM* males.

From our BuzzWatch analyses, we observed central flight patterns only in control males but not in *fruM* males and control females, highlighting that male-specific central flight pattern requires FruM (Fig 1D). However, the FruM-dependent sensory modalities involved remain unknown and require further investigation. In contrast to FruM’s role in regulating male flight patterns, FruM does not determine male-specific WBFs (Fig 1G), suggesting that other sexually dimorphic factors (such as *dsx*) could regulate sex differences in baseline WBFs [46].

Our work provides strong evidence that *fruM* male mating failures are due to a complete loss of phonotaxis and sound-induced abdominal bending behaviors (Fig 1I). Importantly, our results highlight that the loss of phonotaxis in *fruM* males could be partly attributed to defects in mechanical feedback amplification in hearing, sound detection and auditory information processing in mutant ears. This includes the complete loss of male-specific SOs, significantly increased ear tuning frequencies and significantly reduced sound- (pure tone) evoked antennal nerve response in mutant male ears compared to the control males (Fig 2).

Our work demonstrates an important role of FruM in setting the high-frequency sensitivity and selectivity of male ears to female flight sounds at the peripheral sensory level. Based on our definitions of functional auditory organs, however, *fruM* male flagellar ears still display signatures of active hearing and are not deaf. Therefore, the complete loss of phonotaxis and sound-induced abdominal bending behaviors observed in *fruM* males could also be attributed to the additional role of FruM in modulating central processing of auditory cues, which together with FruM’s role in the peripheral auditory system, collectively define the male-specific auditory behaviors.

Unlike *Ae. aegypti*, the antennal ears of *D. melanogaster* are neither anatomically nor functionally sexually dimorphic [47,48]. Here, we identified strong and broad expression of *fru* in mosquito JO neurons (Fig 2). Such intense *fru* expression in JO neurons stands in stark contrast to the sparse expression patterns observed in *D. melanogaster* [49], indicating evolutionary divergence in *fru*’s function and *fru*-dependent sensory modalities involved in male sexual behavior. Given that both species diverged ~250 million years ago, it would be of interest to explore the evolutionary forces driving divergence in antennal ear dimorphic properties, and to understand how *fru* has been recruited to regulate sexually dimorphic neuronal differentiation in anatomically dimorphic mosquito ears [49]. Future work on multi-omics analysis of antennal ears across mosquito and *Drosophila* species could help to address this knowledge gap.

We observed that localization of presynaptic terminals within the JO was altered in *fruM* males compared to control males, with mutant males displaying a lack/reduction of male-type, and gain of female-type, presynaptic terminals (Fig 3). Our work did not elucidate the underlying *fru*-dependent mechanisms that shape sexually dimorphic efferent neuronal arborization patterns within the JO, partially due to our lack of knowledge of the origins of these efferent neuronal populations, i.e., where these efferent neurons descend from. In *D. melanogaster*, *fru* shapes sexually dimorphic neuronal arborization patterns *via* axon guidance, tracking of axon-bound receptors along morphogen gradients of guidance cues, or sex-specific programmed cell death during nervous system development [15,18,50–52]. We hypothesize that defects in these mechanisms could underlie the altered organization of auditory efferent terminals in *fruM* male JOs.

Here, we elucidated key hearing genes that could be under the transcriptional influence of FruM (Fig 4). We identified a female-biased hearing gene, AAEL006647 (*Dlc90F*)*,* that could be part of the mechanotransducer adaptation machinery in female ears, defining female-specific hearing properties. By conducting motif-enrichment and protein-DNA interaction analyses, we found that FruM binds to two different sites in the enhancer region of this gene *via* its zinc-finger DNA-binding domain, suggesting that FruM could directly inhibit the transcription of this gene in male ears (Fig 5). Investigation of AAEL006647, and other promising targets, represents the first step towards elucidating the transcriptional actions of FruM in mosquito ears.

In summary, we propose FruM as the master regulator of male *Ae. aegypti* reproductive behaviors, including male central flight pattern and phonotaxis. FruM defines the male-specific hearing system in mosquitoes, potentially the most important sensory system involved in the early stages of mosquito mating. Our findings demonstrate that FruM directs the performance of the male peripheral auditory organ involved in phonotaxis, thus influencing mating outcomes from the first stage of sound detection and auditory processing. *Via* sophisticated transcriptional networks, FruM modulates neuronal masculinization of male ears by striking a careful balance between promoting male-biased and inhibiting female-biased gene expression programs, enabling male ears to be highly sensitive to female flight sounds. Our work further highlights mosquito flagellar ears as a powerful model to study the actions of *fru*. Finally, we propose *fru* as a new target for genetic-based control strategies aiming to exploit the sex determination pathway to control mosquitoes [53].

## Materials and methods

### Animals used in this study

Unless otherwise noted, all mosquitoes tested were 5–7 days old. Both males and females were provided a diet of sugar water and tested females were not blood-fed. Mosquito lines used in this study were wild-type *Ae. aegypti* (Liverpool strain) and *Ae. aegypti fruM* mutant lines and background controls (Vosshall lab) [21]. All adults tested in this study were virgins. *fruM* males referred to in this study were heteroallelic null mutants generated from crossing *fruitless*^*∆M*^ and *fruitless*^*∆M-tdTomato*^ heterozygous parent lines, enabling definitive screening for homozygous negative mosquitoes as each mutant allele contains a different fluorescent marker. Male and female mosquitoes were used for all experiments described.

### Mosquito rearing

Mosquitoes were reared at 28 °C and 60%–70% relative humidity under 12 h:12 h LD cycles. Larvae were provided with fish food and pupae were sex separated. Adults were provided with 10% glucose water. Blood feeding was conducted *via* a membrane feeding system (Orinno Technology Pte, Singapore).

### Experiment entrainment paradigm

Adult mosquitoes (3–5 days old) were housed in cages or vials and entrained for 2 days under a 12:12 LD regime, except for the phonotaxis assay which adopted a 13:11 LD regime with 1 h of light ramping at the start and end of each light period. Experiments were carried out on entrained mosquitoes starting from the third day of entrainment. All experiments were conducted between ZT11-ZT13, corresponding to the ‘dusk’ phase of entrainment. Mosquitoes had constant access to 10% glucose throughout. All experiments were either carried out in the entrainment incubator set to 22 °C or performed in a temperature-controlled room at 22 °C (±1.5 °C).

### Locomotor activity monitor assay

Individual mosquitoes were aspirated into vials with glucose-soaked cotton wool provided on one end. Vials were then placed in LAM25 Locomotor Activity Monitors (Trikinetics). Following two days of entrainment in an incubator, the number of beam breaks per minute were counted for the next 72 hours. Analyses used the Rethomics package [54].

Sample sizes: 40 control females; 49 control males; 58 *fruM* males

### Video recording of flight behavior

Cages of 30 adult mosquitoes were entrained in an incubator for two days. Flight activity was recorded using a video camera (DMK33UP1300, ARGO; IC Capture Image Acquisition software ver 2.5) on the third day between ZT11.5-ZT12.

### BuzzWatch flight trajectory tracking assay

Groups of 30 adult mosquitoes were entrained in an incubator for two days. On the third day, flight activity between ZT0–ZT12 was recorded using a Raspberry Pi camera (Picamera V2 NOIR). Recordings were analyzed using the BuzzWatch analysis paradigm [24]. Trajectory points were filtered based on maximum flight speed, with points whose calculated speed was greater than 37.5 cm/s being dropped, and the remaining trajectory being split to create a new, separate trajectory. Maximum flight speed used for filtering was based on previous estimates of flight speed using the BuzzWatch system [24]. We estimated a distance of 0.25 mm/pixel for all our recordings.

To create heatmaps for each cage, we used flight position data at dusk (ZT11.5–ZT12). To quantify differences in flight trajectories relative to the cage center between the groups, we investigated three distinct time points; dawn (ZT0–ZT0.5), midday (ZT5.75–ZT6.25) and dusk (ZT11.5–ZT12). The Euclidean distance from each trajectory point to the geometric cage center was calculated for every flight trajectory. We then calculated the radius from the cage center encompassing different percentages of all individual trajectories, with lower values indicating flight closer to the cage center. The r_50_ value of a trajectory thus denotes the radius from the cage center necessary to encompass 50% of that trajectory.

To create Sholl-style plots, we calculated *r*_1_–*r*_100_ (indicating the radii necessary to cover 1%–100% of all individual trajectories) and calculated a weighted average per cage, with this weight calculated based on the length of each trajectory. These weighted averages were then plotted for each cage, with the weighted average r_50_ value also shown for comparisons.

Cage sample sizes: 3 control female cages; 3 control male cages; 3 *fruM* male cages.

Trajectory sample sizes at dusk: 785 control female trajectories; 860 control male trajectories; 489 *fruM* male trajectories.

### Mosquito WBF and fly-by measurements

Cages of 30 adult mosquitoes were entrained in an incubator as described above. During ZT11-ZT12 on the third day, flight tones were recorded by putting a microphone (EK series, Knowles) into the cage. Recordings were made using a Picoscope 2408B and the Picoscope 6.14 software (Pico Technology) at 50 kHz sampling rate. The SimbaR package was used to analyze flight recordings [5] with only flight recordings longer than 150 ms included in the analysis. The median of all analyzed flight recordings was calculated. The number of fly-bys was calculated as the total number of analyzed flight recordings per cage.

Sample sizes: 11 control female cages, 11 control male cages and 11 *fruM* male cages.

### Group-level phonotaxis assay

Male group phonotaxis assays followed published protocols [26,29]. 30 adult male mosquitoes were aspirated into 30 × 30 × 30 cm insect cages (4S3030 Insect Rearing Cages, Bugdorm) and entrained in an incubator for two days. A speaker (FF225WK, Fostex) was placed next to the cage for playback of pure tones between 350 and 850 Hz (in 25 Hz bins). Pure tone files were generated in Audacity 3.0.3 with intensities calibrated to a Particle Velocity Level (PVL) of 1.58 × 10^−4^ ms^−1^, equivalent to 70dB Sound Pressure Level (SPL) [55].

Males were tested for their phonotactic response to pure tones between ZT12.5–ZT13. Each tone lasted for one minute with one minute of silence provided between tones. The order of tone playback was pseudo-randomized between repeats using the sample function in R [56]. A video-camera (DMK33UP1300, ARGO) equipped with a lens (LM5JC1M, Kowa) was placed opposite the speaker to record phonotactic responses. The IC Capture Image Acquisition software (The Imaging Source, version 2.5) was used for all video recordings.

Quantification of phonotactic response was achieved by manual counting of the number of males landing on the netting directly next to the speaker during stimulation. The number of males located in front of the speaker immediately prior to tone onset was subtracted from the number which landed on the netting during tone playback. Counting was conducted without knowledge of tone frequency being played. Response counts were normalized within repeats.

Sample sizes: 2 control male cages; 2 *fru*^*∆M*^/+ male cages; 5 *fruM* male cages.

### Video recording of individual phonotaxis

Four virgin control or *fruM* males were aspirated into a paper cup with glucose-soaked cotton inside. Mosquitoes were entrained for two days in an incubator and tested in a temperature-controlled room set to 22 ± 1.5 °C. Mosquito responses to 400, 450, 470, 500 and 550 Hz pure tone were video-recorded on the third day between ZT11.5–ZT12.

### Identification of target gene expression patterns using published snRNA-seq data

Adult *Ae. aegypti* head snRNA-seq data was downloaded from Goldman and colleagues, 2025 [27] and expression patterns of target genes were plotted using the Seurat package in R [57]. Male and female cells were labeled based on the sex of the samples and putative JO neuron clusters were identified based on filtering for cells with *inactive* (AAEL020482) expression.

### *fru* splicing analysis

Differential splicing analysis of genes was performed using DEXSeq [58] in R. *Ae. aegypti* GFF file downloaded from VectorBase [59] (68th release) was used to create a flattened annotation. BAM files containing the aligned reads of *Ae. aegypti* male and female pedicels [28] were mapped to the flattened annotation and exon usage counts for *fru* (AAEL024283) were obtained using the featureCounts function in Rsubread package [60].

### JO immunofluorescence

Individual mosquitoes were sedated on ice before their heads were removed and fixed for 1 h in 4% paraformaldehyde (PFA) in phosphate-buffered saline (PBS) with 0.25% Triton X-100 (PBT). Fixed heads were embedded in 10% agarose solution (Agarose S, 318-01195) and kept at 4 °C for 10 min to solidify the agarose blocks. Blocks were then fixed overnight in 4% PFA at 4 °C.

Blocks were washed in 100% methanol for 10 min, transferred to PBS for 30 min and sectioned into 40 μm slices using a vibratome (Leica VT1200S). Sections were washed in 0.5% PBT three times at room temperature and blocked in 10% normal goat serum (NGS) (Vector Laboratories)/0.5% PBT for 1 h. Sections were incubated at 4 °C with primary antibodies in 10% NGS/0.5% PBT for three days.

Sections were washed three times with 0.5% PBT before a two-day incubation at 4 °C with secondary antibodies in 10% NGS/0.5% PBT. After three washes with 0.5% PBT and one wash with PBS, sections were mounted on microscope slides and imaged with a laser-scanning confocal microscope (FV3000, Olympus) using 20× air objective (UPlanSApo, NA = 0.75) lens.

Primary antibodies used: anti-SYNORF1 3C11 monoclonal antibody (AB_528479, 1:30, Developmental Studies Hybridoma Bank (DSHB), University of Iowa); anti-SAP47 nc46 monoclonal antibody (AB_2617213: 1:30; DSHB); anti-DsRed polyclonal antibody (AB_10013483, 1:300, Takara Bio).

Secondary antibodies used: Alexa Fluor 488-conjugated anti-mouse IgG (A-11029, 1:300, ThermoFisher), Alexa Fluor 555-conjugated anti-rabbit IgG (A-21428, 1:300, ThermoFisher) and Cy3-conjugated goat anti-horseradish peroxidase (anti-HRP, AB_2338959, 1:150, Jackson Immuno Research).

### Brain immunofluorescence

Mosquito heads were fixed in 4% PFA in 0.25% PBT for 3 hours. Heads were washed twice with PBS before brains were dissected. Following dissection, brains were washed six times in 1% PBT at room temperature before permeabilization with 2% NGS in 4% PBT. After two days of incubation at 4 °C, brains were washed six times with 1% PBT at room temperature then incubated in 1% PBT plus 2% NGS with primary antibodies for 2 days at 4 °C. Brains were washed six times with 1% PBT at room temperature and incubated in 1% PBT plus 2% NGS with secondary antibodies for 2 days.

Finally, brains were washed six times with 1% PBT at room temperature and mounted in 80% glycerol in PBS. Samples were imaged with a laser-scanning confocal microscope (FV3000, Olympus) using a silicone-oil immersion 30× Plan-Apochromat objective lenses (UPlanSApo, NA = 1.05).

Primary antibodies used: anti-Brp (nc82) (AB_2314866, 1:30, DSHB) and anti-DsRed polyclonal antibody (AB_10013483, 1:300, Takara Bio).

Secondary antibodies used: Alexa Fluor 488-conjugated anti-mouse IgG (A-11029, 1:300, ThermoFisher), Alexa Fluor 555-conjugated anti-rabbit IgG (A-21428, 1:300, ThermoFisher).

### Mosquito preparation for laser Doppler vibrometry and electrophysiology

Mosquito preparation followed previous protocols [12,26]. Mosquitoes were sedated on ice and glued to a plastic rod using blue-light-curable glue (Norland Products, 81). Only the mosquito’s right flagellum was free to move after gluing. Mounted mosquitoes were tested in a temperature-controlled room set to 22 ± 1.5 °C. The rod holding the mosquito was inserted into a micromanipulator (MM-3, Narishige Instruments, Japan) on a vibration isolation table. All vibrometry recordings were performed using a laser Doppler vibrometer (Vibroflex, Polytec) and the VibSoft software (Polytec).

For electrophysiology recordings, a sharpened tungsten reference electrode was inserted into the mosquito thorax to enable charging to −30 V relative to ground. Electrostatic actuators were positioned around the flagellum to enable provision of electrostatic stimulation. A tungsten recording electrode was inserted into the base of the pedicel to allow for antennal nerve recordings. The laser Doppler vibrometer was focused on the first flagellomere from the tip of the flagellum to enable simultaneous measurements of flagellar displacement. All electrophysiology recordings were collected using the Spike2 software (ver 10.08, CED), with stimuli created in Spike2.

### Laser Doppler vibrometry: Recordings

Stimulated recordings of the mosquito flagellum in the active state were made by measuring the mechanical vibrational response of the mosquito flagellum to white noise (1–2,000 Hz, Audacity 3.0.3) playback provided by a speaker (FF85WK, Fostex) placed 1.5 cm from the mosquito. The speaker was calibrated to a PVL of 1.58 × 10^−4^ ms^−1^, equivalent to 70 dB SPL [55].

For unstimulated recordings, prior to measuring the passive state free-fluctuations of mosquito flagellum, an active-state free-fluctuation recording was first obtained. Mosquitoes were then exposed to CO_2_ in a sealed chamber; depriving mosquitoes of oxygen leads to a (reversible) cessation of metabolic activity. An unstimulated recording of the sedated mosquito flagellar ear was immediately taken once CO_2_ delivery was stopped.

### Laser Doppler vibrometry: Data analysis

Fast Fourier transformation of raw time-domain data was conducted using the VibSoft software (Polytec) between 1 and 10,000 Hz. Values below 125 and above 1,000 Hz were excluded from further analyses due to noise. A forced damped harmonic oscillator function was fit to all transformed data using the lme4 package [61] in R:


$$\begin{array}{c} \dot{X}(\omega )=\frac{\omega .\frac{F_{\text{0}}}{m}}{\sqrt{\left({\left({\omega_{\text{0}}^{\text{2}}-\omega }^{\text{2}}\right)}^{\text{2}}+{\left(\omega .\frac{\omega_{\text{0}}}{Q}\right)}^{\text{2}}\right)}} \end{array}$$ (1)

With *F*_0_ = external force strength, *m* = flagellar apparent mass, *ω* = angular frequency, *ω*_0_ = natural angular frequency and *Q* = quality factor = *mω*_0_/*γ* (*γ* = damping constant).

Fitting this function facilitated calculation of the natural angular frequency (*ω*_0_) for each recording, enabling calculation of the flagellar ear mechanical tuning frequency, *f*_0_, derived from *f*_0_ = *ω*_0_/2*π*.

Sample sizes: 15 control females; 19 control males; 12 *fruM* males.

### Electrophysiology: Recordings

Prior to frequency sweep stimulation, a calibrated force-step stimulus was provided to each mosquito to calibrate the flagellum displacement to approximately ±2.5μm. Stimulation paradigms followed published protocols [12,26].

For frequency sweep experiments, sets of sweep stimuli, including one-second-long chirps of linearly increasing (forward, 1–1,000 Hz) or decreasing (backward, 1,000–1 Hz) frequency, were provided whilst the flagellar displacement (100 kHz sampling rate) and antennal nerve response (20 kHz sampling rate) were recorded in Spike2. Laser quality data (10 kHz sampling rate) was recorded to facilitate analyses.

For pure tone-based threshold experiments, 500 ms pure tone stimuli were provided at different levels of attenuation. Each series of pure tones per attenuation consisted of four sets of phasic and anti-phasic stimuli per frequency. Between each stimulus frequency, 500 ms of silence was provided, with one minute of silence between attenuations. Range of pure tones: 75–300 Hz for control females; 100–575 Hz for control and *fruM* males.

Mosquito flagella were exposed to a total of 40 sets of force step stimulation of seven different amplitudes. Each set of force step stimulation consisted of 200 ms positive force step and 200 ms negative force step with a 200 ms silence between directions. A 400 ms of silence was provided between two sequential sets of stimulation.

### Electrophysiology: Sweep analysis

For flagellar mechanical tuning analyses, laser quality data recorded as zero were excluded. Channel processes were applied to the laser channel data: a DC remove function (0.01 s time constant); rectification; a smooth function (0.0005 s time constant); a slope function (0.1 s time constant). This enabled identification of when the channel slope was equal to zero, i.e., the time of maximum flagellar vibration. The corresponding stimulus frequency was taken as the peak ear mechanical tuning frequency.

For electrical tuning analyses of antennal nerve compound action potential responses, nerve responses to sequential phasic and anti-phasic stimuli were averaged to cancel out artefacts resulting from electrostatic actuation. The resulting nerve signal had the following processes applied: a DC remove function (0.01 s time constant); rectification; a smooth function (0.0005 s time constant); a slope function (0.1 s time constant). Calculation of the time when the slope equaled zero enabled estimation of the corresponding stimulus frequency, referred to as the peak ear electrical tuning frequency.

For peak mechanical tuning frequency calculations, the average of consecutive phasic and anti-phasic sweeps of the same orientation was taken. The median of all these averages was taken as the peak mechanical tuning for that individual. For the peak electrical tuning frequency, the average between forward and backward sweeps was computed and the median was taken across averages (S11A Fig).

Sample sizes: 19 control females; 17 control males; 13 *fruM* males.

### Electrophysiology: Pure tone analysis

Nerve responses to sequential phasic and anti-phasic stimuli were averaged to cancel artefacts in the nerve channel. A DC remove (0.01s time constant) was applied to the resulting averaged nerve channel, followed by rectification. The Area Under the Curve (AUC) was then calculated for the 250 ms prior to stimulus onset and the last 250 ms of stimulus playback, for all stimuli. The threshold voltage for each stimulus frequency was defined as the voltage at which the AUC during stimulus playback was significantly greater than the AUC prior to playback, and for which all stimuli of greater voltage amplitude also elicited a significant nerve response. The minima threshold frequency was determined as the frequency that elicited significant antennal nerve responses at the threshold voltage. Nerve AUC response magnitudes were normalized to stimulus voltage for plotting. Maximum nerve phasic responses were determined as the peak nerve response estimated from loess fits of the nerve phasic response profile for each individual mosquito to pure tones provided at the largest stimulus voltage (S12A Fig).

Sample sizes: 17 control females; 17 control males; 16 *fruM* males.

### Electrophysiology: Force-step stimulation analysis

Flagellar displacements in response to force-step stimulation were calculated by measuring the difference in flagellar displacements between the 5 ms average displacement at 100 ms after stimulus onset and the 5 ms average displacement immediately before stimulus onset. Nerve responses were calculated by subtracting 50 ms of average nerve baseline at 50 ms before stimulus onset from the nerve minima obtained within 2.5 ms after stimulus onset. Maximum nerve tonic responses were quantified as the largest nerve response recorded for each individual mosquito across different step magnitudes (S14A Fig).

Sample sizes: 17 control females; 17 control males; 15 *fruM* males.

### JO width measurements

JO width measurements were calculated as the longest distance between the outer somata of both sides of the JO. Image stacks stained with HRP were used for measurements in ImageJ (version 1.53q) [62]. Only JOs in which the basal plate could be clearly identified were analyzed.

Sample sizes: 14 control females; 14 control males; 13 *fruM* males.

### JO synaptic punctae quantification

Image stacks stained with HRP and 3C11 antibodies were used for quantification. The SynBot analysis software was utilized to estimate the number of punctae in both ears of mosquitoes from each group [63]. Only JOs in which the basal plate could be clearly identified were analyzed. Two regions of each JO were selected, representing the regions corresponding to Type III (somata) and Type VI (inner JO dendritic cilia) punctae.

Sample sizes for type III terminals: 6 control female JOs; 8 control male JOs; 4 *fruM* male JOs.

Sample sizes for type VI terminals: 6 control female JOs; 6 control male JOs; 4 *fruM* male JOs.

### cAMP and TeNT injection experiments

Mosquitoes were mounted as described above for vibrometry experiments. A free fluctuation recording was taken using the VibSoft software (Polytec) to act as a baseline prior to injection. Following this, compounds were injected into the mosquito and further recordings were made.

For cAMP experiments, either 3mM 8-Bromoadenosine 3,5-cyclic monophosphate sodium salt (8-Br-cAMP; 76939-46-3, Sigma), or Ringer’s control [12] solution was injected into the mosquito thorax. Recordings were made 5, 10, 15 and 20 min following injection.

For TeNT injection, 2μM TeNT (T3194, Sigma) or Ringer’s solution was injected into the mosquito thorax. Recordings were made every 5 min for 60 min following injection.

Recordings were analyzed as described above to extract the peak mechanical tuning frequency, enabling calculation of frequency change (ΔFrequency) at each recording time point compared to pre-injection baseline. The largest ΔFrequency within an individual recording was taken as the maximum ΔFrequency.

Fluctuation power was calculated by first converting data to the displacement squared domain, then estimated using the AUC function in R [64]. By dividing the AUC for each post-injection time point by the pre-injection baseline, the maximum AUC ratio following injection was calculated.

Sample sizes for 8-Br-cAMP/Ringer injections: 10/7 control females; 8/8 control males; 8/8 *fruM* males.

Sample sizes for TeNT/Ringer injections: 3/3 control females; 3/3 control males; 4/3 *fruM* males.

### Transcriptomics sample preparation

Five to six days old mosquitoes were flash-frozen in liquid nitrogen at ZT12. Pedicels were dissected on ice in RNAiso Plus (9109, Takara Bio). Tissues were homogenized using a handheld homogenizer (BT LabSystems). RNAiso Plus was added to the homogenate to make up to a final volume of 1 mL and incubated at room temperature for 15 min prior to being added with 200 µL of chloroform (Kanto Chemical Co.). Samples were well mixed followed by a 15-min incubation at room temperature. Samples were centrifuged at 12,000*g* for 15 min at 4 °C. After centrifugation, supernatant was transferred to a fresh tube followed by the addition of 0.5 mL of ice-cold 2-propanol (Sigma) to each sample. Samples were mixed well before being kept at −20 °C for 30 min.

Samples were thawed and centrifuged at 12,000*g* for 15 min at 4 °C, supernatant was discarded, and the RNA pellets were washed with 1 mL of 75% ethanol (Sigma) solution twice. RNA pellets were dried and dissolved in DEPC water (Nacalai Tesque). RNA quality was assessed using Nanodrop (ThermoFisher Scientific). Five biological repeats for each group were submitted to the Center for Gene Research at Nagoya University for library preparation.

### Transcriptomics: Library preparation, read alignment and differential expression analysis

*Ae. aegypti* L5 genome fasta file was downloaded from VectorBase (68th release) for read alignment. Single-end reads were aligned to the genome using the align function in Rsubread in R. By using the *Ae. aegypti* GFF file downloaded from VectorBase (68th release), gene counts of aligned reads were obtained using the featureCounts function provided in Rsubread package.

Gene annotation was conducted by retrieving *Ae. aegypti* gene relevant information from the EnzemblMetazoa using the biomaRt package [65] in R. Differential expression analysis was carried out using DESeq2 package [66] in R by setting a fold-change threshold of 1 and FDR cutoff <0.1. DESeq2 normalized counts were exported and used as RNA expression counts in heatmaps presented.

To further restrict the differentially expressed gene lists to pedicel-relevant genes, we applied a filter criterion for all analyzed gene lists to only include genes that showed higher summed read counts in the pedicel than in the head of either sex, by using a previously published pedicel and head transcriptomic datasets of male and female *Ae. aegypti* mosquitoes [28].

### *Aedes aegypti* and *Drosophila melanogaster* gene ortholog conversion

Gene orthologs between *Ae. aegypti* and *D. melanogaster* were downloaded from OMA Browser [67] and EnsemblMetazoa [68] (downloaded on 7th January 2025). EnzemblMetazoa ortholog gene list was fetched *via* the biomaRt package in R [65]. Duplicates from the two downloaded gene lists were removed. The ortholog gene list file was used to improve *Ae. aegypti* gene annotation.

### Improve *Ae. aegypti* GAF file for Gene Ontology enrichment analysis

Complete proteome of *Ae. aegypti* was downloaded from NCBI (downloaded on 7th January 2025) and subjected to GO annotations using PANNZER2 [69]. PANNZER2 results were combined with GAF file downloaded from VectorBase followed by removing duplicates.

### Gene Ontology enrichment analysis

The improved GAF file was used to run GO enrichment analysis using g:Profiler [70]. Custom GMT file used in this study can be found using the following g:Profiler token: gp__E7yL_EL9T_8t8. The SCS threshold algorithm was selected for significance testing with a significance threshold of 0.05 applied.

### Motif enrichment analysis

Motif enrichment analysis on the 1kbp enhancer sequences upstream of translational start site of target genes was conducted using findMotifs.pl (-fasta) from HOMER [71]. Background fasta files used to run the enrichment analysis included the 1kbp enhancer sequences of all genes in the genome that were not part of the target gene groups. Enhancer sequences were retrieved using the biomaRt::getSequence [65] function in R. Motifs of interest were further scanned in the enhancer sequences of other gene groups to determine their motif occurrences and positions using the findMotifs.pl (-find). Automated motif clustering was performed using universalmotif package in R [72].

### AlphaFold3 prediction of protein-DNA interactions

The C_2_H_2_ zinc-finger B protein domain (C_2_H_2_-Zn-B) of FruM was used as the protein query to predict interactions with target DNA motifs using AlphaFold3 [42,43]. Two Zn^2+^ ions were also included to model the interactions. Predicted protein-DNA models were downloaded from AlphaFold3 and analyzed in PyMol [73]. The pairwise distance between interacting amino acid residues and motif base pair residues was extracted using the pairwisedistances.py script available on PyMolWiki. Minimal pairwise distance threshold of <4.5 Å was determined based on prior studies [74–76]. Single base pair mutagenesis was conducted *via* base pair transversion (C ↔ A or T ↔ G). Amino acid substitutions at alpha helixes were done by replacing target amino acid with proline to introduce kink in the alpha helix structure.

### Protein purification

Protein-coding sequences for the zinc-finger domain (Zn-B) of FruM and the truncated FruM lacking the zinc-finger domain were fused with a 6xHis-MBP-tag at the N-terminus and cloned into a p15TV-L plasmid (Addgene, GenBank ID: EF456736.1). The plasmid was then transformed into Rosetta-gami 2 (DE3) (Sigma-Aldrich) to induce protein expression. Bacterial cells were grown in LB medium supplemented with 100 μM ZnSO_4_ at 37 °C and 180 rpm until the optical density OD600 reached 0.5–0.8. Protein expression was induced by adding 100 μM of IPTG (Sigma-Aldrich) and shaking at 18 °C overnight.

After induction, cells were harvested and resuspended in HEPES and glycerol (final concentration of 100 mM HEPES (pH 7.5), 10% glycerol). Cells were then lysed by sonication, and the lysate was centrifuged at 10,000 rpm for 30 min at 4 °C. Supernatant was transferred to a fresh tube and Ni-NTA resin (Profinity IMAC Resin, Ni-charged) pre-equilibrated with extraction buffer (100 mM HEPES (pH 7.5), 150 mM NaCl, 100 μM ZnSO_4_ and 10% glycerol) added. After 30 min of pull-down using a rotor at 4 °C, the beads were collected by centrifugation at 4,000 rpm for 1 min. Supernatant was discarded and beads transferred to an Eppendorf before washing three times with wash buffer (extraction buffer + 30 mM imidazole).

The protein was eluted in elution buffer (extraction buffer + 150 mM imidazole). Desalting and buffer exchange (20 mM HEPES (pH 7.5), 30 mM NaCl, 100 μM ZnSO_4_) was performed using HiTrap Desalting columns with Sephadex G-25 resin. Finally, protein was aliquoted and stored at −80 °C.

### Electrophoretic Mobility Shift Assay

DNA probes for EMSA were labeled with Fluorescein-5-maleimide (Tokyo Chemical Industry) using 5′ EndTag DNA/RNA Labeling Kit (Vector Labs). DNA binding reaction contained 350 ng of labeled DNA, 7 µg of protein, 10× binding buffer (1× binding buffer: 10 mM (NH_4_)_2_SO_4_, 0.2% Tween20, 5% glycerol, 1 mM DTT and 1 µg BSA) and exchange buffer in total volume of 20 µL.

The reactions were incubated in darkness at room temperature for 1 h, followed by gel electrophoresis using 1.5% agarose gel (in 0.5× TB). 3 µL of 30% glycerol was added before gel-loading. Gel electrophoresis was performed at 150 V for 20 min and the resulting gel was viewed using a gel-viewer (Atto).

### Quantification and statistical analysis

*P* < 0.05 (prior to correction) was set as the significance level for all statistical testing. Two-sided statistical tests were used throughout.

Shapiro–Wilk tests of normality were first used to test for normality for all flight assay, functional assay and JO width data. Flight assay and JO width data were found to be normally distributed, so pairwise *t*-tests with BH corrections were used for statistical testing. Functional datasets including sweep data, white noise stimulation and sedation vibrometry recordings, as well as static-deflections-induced antennal nerve responses were also found to be normally distributed, as such, pairwise *t*-tests with BH correction were used.

Functional datasets found to be non-normally distributed were LAM data, BuzzWatch *r*_50_ quantification and threshold pure tone stimulation data. These datasets were tested for significance using pairwise Wilcoxon tests with BH corrections. The vibrometry datasets for the injection of Ringer and cAMP were tested using Wilcoxon tests.

**Table 2:** Parameters associated with vibrometry and electrophysiology for all groups tested. Sweep data provided as means  ±  standard deviations, pure tone data provided as median values ±standard errors. Numbers in brackets refer to sample sizes for each experiment.

Group	Mechanical tuning frequency – Sweeps (Hz)	Electrical tuning frequency – Sweeps (Hz)	Threshold electrical tuning frequency – Pure tones (Hz)	Minima voltage – Pure tones (V)
Control female	245.25 ± 23.05 (*n* = 19)	175.98 ± 22.35 (*n* = 19)	199.62 ± 4.89 (*n* = 17)	1.82 × 10^−2^ ± 7.95 × 10^−4^ (*n* = 17)
Control male	405.42 ± 44.66 (*n* = 17)	297.18 ± 45.00 (*n* = 17)	299.50 ± 9.80 (*n* = 17)	1.60 × 10^−2^ ± 1.57 × 10^−4^ (*n* = 17)
*fruM* male	495.22 ± 46.53 (*n* = 13)	358.60 ± 21.45 (*n* = 13)	345.85 ± 8.19 (*n* = 16)	1.76 × 10^−2^ ± 4.75 × 10^−4^ (*n* = 16)

## Supporting information

S1 Fig Loss of FruM alters male locomotor activity. **(A)** Locomotor Activity monitor diagram. **(B)** Summed activity counts per hour for control females, control males and *fruM* males over three days of LD activity. Yellow shaded region represents lights on, gray shaded region represents lights off. Sample sizes: 40 control females; 49 control males; 58 *fruM* males. **(C)** Summed beam breaks over 3 days for control female, control male and *fruM* male groups during ZT11–ZT12. Individual points represent summed beam breaks for individual mosquitoes. Solid lines represent medians and standard errors. See S1 Table for *p* values. n.s., *p* > 0.05, * *p* < 0.05, ** *p* < 0.01, *** *p* < 0.001, pairwise Wilcoxon tests with BH correction. Sample sizes: 40 control females; 49 control males; 58 fruM males. The data underlying this figure can be found at https://doi.org/10.17632/dtscz72px6.1.
(TIF)

S2 Fig Individual cage position heatmaps from BuzzWatch recordings at dusk. **(A)** Inside of the BuzzWatch recording chamber. Edge of the cage highlighted in yellow. **(B)** Heatmaps of control female (top), control male (middle) and *fruM* male (bottom) pixel occupancy across three repeats during ZT11.5–ZT12. Each repeat contained 30 mosquitoes.
(TIF)

S3 Fig Example trajectories from control female BuzzWatch recordings. **(A)** 10 example trajectories from control female BuzzWatch recordings at dawn (ZT0–ZT0.5). **(B)** 10 example trajectories from control female BuzzWatch recordings at midday (ZT5.75–ZT6.25). **(C)** 10 example trajectories from control female BuzzWatch recordings at dusk (ZT11.5–ZT12).
(TIF)

S4 Fig Example trajectories from control male BuzzWatch recordings. **(A)** 10 example trajectories from control male BuzzWatch recordings at dawn (ZT0–ZT0.5). **(B)** 10 example trajectories from control male BuzzWatch recordings at midday (ZT5.75–ZT6.25). **(C)** 10 example trajectories from control male BuzzWatch recordings at dusk (ZT11.5–ZT12).
(TIF)

S5 Fig Example trajectories from *fruM* male BuzzWatch recordings. **(A)** 10 example trajectories from *fruM* male BuzzWatch recordings at dawn (ZT0–ZT0.5). **(B)** 10 example trajectories from *fruM* male BuzzWatch recordings at midday (ZT5.75–ZT6.25). **(C)** 10 example trajectories from *fruM* male BuzzWatch recordings at dusk (ZT11.5–ZT12).
(TIF)

S6 Fig Differences in r_50_ across groups and circadian time. **(A)** Changes in cumulative percentage of trajectories within increasing radius (in number of pixels) of cage center for each individual repeat. Values calculated at dawn (ZT0–ZT0.5), midday (ZT5.75–ZT6.25) and dusk (ZT11.5–ZT12). Each repeat contained 30 mosquitoes. Weighted averages calculated across all trajectories, with flight length used for weighing. **(B)** *r*_50_ values calculated for each repeat across three timepoints. *r*_50_ defined as circle radius (in number of pixels) from cage center necessary to contain 50% of all trajectories. Relatively smaller *r*_50_ values thus indicate more central flight pattern. Weighted averages calculated across all trajectories, with flight length used for weighing. Solid black line represents median and the connected lines between data points indicate data points obtained from the same repeat (cage). The data underlying this figure can be found at https://doi.org/10.17632/dtscz72px6.1.
(TIF)

S7 Fig Heterozygous *fruM* mutant males demonstrate phonotaxis. **(A)** Normalized responses for *fruitless*^*∆M*^/+ male cages to different frequencies of sound (350–850 Hz in 25 Hz steps). Individual points represent normalized responses for individual cages. Solid lines show average response between repeats. Responses normalized within a repeat. Sample size: 2 *fru*^*∆M*^/+ male cages. The data underlying this figure can be found at https://doi.org/10.17632/dtscz72px6.1.
(TIF)

S8 Fig *fru* expression in male JO. **(A)** Heatmap of exon usage counts of m, f and c1 exons in control female and control male pedicels as identified from reanalysis of published adult male and female *Ae. aegypti* pedicel transcriptome data [28]. **(B)** Split-channel images of *fru* expression in *fru*^*ΔM-tdTomato*^/+ male JO shown in Fig 2C. Scale bar, 100 μm. Maximum intensity projections of JOs stained with anti-tdTomato (yellow) and anti-HRP (blue). The data underlying this figure can be found at https://doi.org/10.17632/dtscz72px6.1.
(TIF)

S9 Fig *fru* expression in male and female AMMC. **(A)** *fru* expression in *fru*^*ΔM-tdTomato*^/+ male brain focusing on antennal mechanosensory and motor center (AMMC) (left) as well as a whole-brain image (right). Scale bar, 100 μm. Maximum intensity projections of brains stained with anti-tdTomato (yellow) and anti-Brp (nc82, blue). Outlined regions in left images: red indicates AMMC, magenta indicates antennal lobe (AL) and green indicates JO neuron axon bundle. All images are from the same brain with different layer coverage. **(B)** *fru* expression in *fru*^*ΔM-tdTomato*^/+ female brain focusing on AMMC (left) as well as a whole-brain image (right). Scale bar, 100 μm. Maximum intensity projections of brains stained with anti-tdTomato (yellow) and anti-Brp (nc82, blue). Outlined regions in left images: red indicates AMMC, magenta indicates antennal lobe (AL) and green indicates JO neuron axon bundle. All images are from the same brain with different layer coverage. The data underlying this figure can be found at https://doi.org/10.17632/dtscz72px6.1.
(TIF)

S10 Fig Loss of FruM alters male hearing function. **(A)** Difference between active, white noise-stimulated mechanical tuning frequencies (Fig 2E) and sedated, unstimulated mechanical tuning frequencies (Fig 2F) of control females, control males and *fruM* males. Individual points represent measurements from individual mosquitoes. Solid lines represent means and standard deviations. See S7 Table. *** *p* < 0.001, pairwise *t*-tests with BH correction. Sample sizes: 15 control females; 19 control males; 12 *fruM* males. **(B)** Power spectral densities from harmonic oscillator fits to unstimulated free fluctuations of control female, control male and *fruM* male flagellar ears in active and passive states. Thick lines represent fits generated based on median fit parameters. Gray lines represent passive system fits, colored lines represent active system fits. Sample sizes: 15 control females; 19 control males; 12 *fruM* males. **(C)** Histogram of control female WBFs (red, Fig 1G) compared to control male (blue) and *fruM* male (orange) mechanical tuning frequencies in active, stimulated (sound, Fig 2E) and active, unstimulated (no sound, S10B Fig) states. Colored bars represent mechanical tuning frequencies across sound intensity gradient, from left (Sound/stimulated) to right (No sound/unstimulated). The data underlying this figure can be found at https://doi.org/10.17632/dtscz72px6.1.
(TIF)

S11 Fig Electrophysiology recording analysis paradigm for sweep stimuli. **(A)** Analysis paradigm for sweep stimulus experiments. Flagellar displacements (middle row) and compound action potential responses (bottom row) to sweep simulation (top row) were simultaneously measured. Analysis of nerve data required duplication of the nerve channel, followed by timeshifting and averaging to remove artefacts arising from the use of electrostatic actuation to provide the stimulus. Laser and (processed) nerve data were rectified and smoothed, before a slope function was applied. Identification of points at which the slope equaled zero enabled calculation of the maximum mechanical and electrical responses.
(TIF)

S12 Fig Electrophysiology recording analysis paradigm for pure tone stimuli. **(A)** Analysis paradigm for pure tone stimulus experiments. Compound action potential responses (bottom row) to stimulation (top row) were recorded. Analysis of the nerve data required duplication of the nerve channel, followed by timeshifting and averaging to remove artefacts arising from the use of electrostatic actuation to provide the stimulus. The Area Under Curve (AUC) in the 250 ms before stimulus onset and before stimulus offset were then calculated.
(TIF)

S13 Fig Antennal nerve responses to pure tone stimulation. **(A)** Example neural responses from control female, control male and *fruM* mutant males to pure tone stimulation. Frequency of tone for each group selected based on frequency that elicited maximum neural response. **(B)** Threshold electrical responses of control female, control male and *fruM* male ears across a range of pure tone frequencies. Bars represent mean minimum voltage necessary to elicit significant response per frequency across all repeats. Individual points represent individual mosquito data. Sample sizes: 17 control females; 17 control males; 16 *fruM* males. The data underlying this figure can be found at https://doi.org/10.17632/dtscz72px6.1.
(TIF)

S14 Fig Electrophysiology recording analysis paradigm for step stimuli. **(A)** Analysis paradigm for step stimulus experiments. Flagellar displacements (middle row) and compound action potential responses (bottom row) to step simulation (top row) were simultaneously measured. The magnitude of the maximal compound action potential response to each stimulus was calculated, in addition to the flagellar displacement relative to baseline following stabilization of flagellar movement.
(TIF)

S15 Fig Loss of FruM alters localization of presynaptic terminals in male JOs. **(A)** 3C11 staining within JO. Split-channel images of JO IHC shown in Fig 3D. Scale bar, 100 μm. Maximum intensity projections of JOs stained with anti-SYNORF1 (3C11, green) (top). Close-up views of type III (somata), type V (basal plate) and type VI (inner JO dendritic cilia) efferent presynaptic terminal sites taken from JO images shown in Fig 3D. Scale bar, 20 μm. JOs stained with anti-SYNORF1 (3C11, green) and anti-HRP (magenta). **(B)** nc46 staining within JO. Split-channel images of JO IHC shown in Fig 3E. Scale bar, 100μm. Maximum intensity projections of JOs stained with anti-SAP47 (nc46, blue) (top). Close-up view of type III (somata), type V (basal plate) and type VI (inner JO dendritic cilia) efferent presynaptic terminal sites taken from JO images shown in Fig 3E. Scale bar, 20 μm. JOs stained with anti-SAP47 (nc46, blue) and anti-HRP (magenta). The data underlying this figure can be found at https://doi.org/10.17632/dtscz72px6.1.
(TIF)

S16 Fig Quantification of altered localization of presynaptic terminals in the JOs. **(A)** Quantification of the abundance of type III (left) and type VI (right) presynaptic terminals in control female, control male and *fruM* male JOs. Presynaptic terminals were visualized via 3C11 staining and quantified using SynBot. Solid lines represent medians and standard errors. Individual points represent estimated abundances obtained for individual JOs. Sample sizes for type III terminals: 6 control female JOs; 8 control male JOs; 4 *fruM* male JOs. Sample sizes for type VI terminals: 6 control female JOs; 6 control male JOs; 4 *fruM* male JOs. The data underlying this figure can be found at https://doi.org/10.17632/dtscz72px6.1.
(TIF)

S17 Fig Changes in mosquito hearing function following compound injection. **(A)** Power spectral densities from harmonic oscillator fits to free fluctuations of control male flagellar ear before (light blue) and after (dark blue) TeNT injection. Sample size: 3 control males. **(B)** Maximum change in mechanical tuning frequency for vibrometry recordings taken after compound injection (Ringer or cAMP) relative to pre-injection baseline for control females, control males and *fruM* males. Individual points represent maximum changes in mechanical tuning after compound injection (Ringer or cAMP) compared to pre-injection baseline for individual mosquitoes. Solid lines show medians and standard errors. See S17 and S19 Tables for maximum Δfrequency values and *p* values. * *p* < 0.05; ** *p* < 0.01, Wilcoxon tests. Sample sizes for Ringer and cAMP injections: 7/10 control females; 8/8 control males; 8/8 *fruM* males. The data underlying this figure can be found at https://doi.org/10.17632/dtscz72px6.1.
(TIF)

S18 Fig Changes in mechanical tuning following cAMP injection. **(A)** Power spectral densities from harmonic oscillator fits to free fluctuations of control female flagellar ear before (pink) and after (red) ringer injection. Sample size: 7 control females. **(B)** Power spectral densities from harmonic oscillator fits to free fluctuations of control female flagellar ear before (pink) and after (red) cAMP injection. Sample size: 10 control females. **(C)** Power spectral densities from harmonic oscillator fits to free fluctuations of control male flagellar ear before (light blue) and after (dark blue) ringer injection. Sample size: 8 control males. **(D)** Power spectral densities from harmonic oscillator fits to free fluctuations of control male flagellar ear before (light blue) and after (dark blue) cAMP injection. Sample size: 8 control males. **(E)** Power spectral densities from harmonic oscillator fits to free fluctuations of *fruM* male flagellar ear before (light orange) and after (dark orange) ringer injection. Sample size: 8 *fruM* males. **(F)** Power spectral densities from harmonic oscillator fits to free fluctuations of *fruM* male flagellar ear before (light orange) and after (dark orange) cAMP injection. Sample size: 8 *fruM* males. The data underlying this figure can be found at https://doi.org/10.17632/dtscz72px6.1.
(TIF)

S19 Fig Gene ontology enrichment analysis. **(A)** Network of GO enrichment terms significantly enriched in genes significantly upregulated in *fruM* males compared to control males. Size and color of the GO term nodes represent the number of genes identified under each GO term. Thickness of the edges represents the number of shared genes between two GO term nodes. **(B)** Network of GO enrichment terms significantly enriched in genes significantly downregulated in *fruM* males compared to control males. Size and color of the GO term nodes represent the number of genes identified under each GO term. Thickness of the edges represents the number of shared genes between two GO term nodes.
(TIF)

S20 Fig Correlation plots of fold change in gene expression between comparison groups. **(A)** Log_2_ fold change in control females and *fruM* males compared to control males of genes potentially linked to hearing function in s_142_. **(B)** Log_2_ fold change in control males compared to control females and *fruM* males of genes potentially linked to hearing function in s_175_. (**C**) Log_2_ fold change in control males compared to control females and *fruM* males compared to control males of genes potentially linked to hearing function in s_257_. The data underlying this figure can be found at https://doi.org/10.17632/dtscz72px6.1.
(TIF)

S21 Fig Identification of putative FruM-binding motifs. **(A)** Pairwise distance analysis between Zn-B of FruM and FruM motif cluster members. All interactions <4.5 Å between Zn-B and each nucleotide position plotted. Red shaded boxplots are nucleotide positions consistently showing interactions with Zn-B across all three models. **(B)** Heatmap of the minimal distance (Å) between consensus or single-base-mutated DNA motif and the indicated amino acid residue of Zn-B. Only interactions with minimal distance <4.5 Å were plotted (left). AlphaFold3 predicted protein-DNA complexes between Zn-B of FruM and the single base-mutated DNA motif for nucleotide position 2, 3 and 5 (right). **(C)** Heatmap of the minimal distance (Å) between the indicated amino acid positions of native Zn-B or single proline-substituted mutant protein and the consensus DNA motif. Only interactions with minimal distance <4.5 Å were plotted (left). AlphaFold3 predicted protein-DNA complexes between consensus DNA motif and single proline-substituted mutant protein for amino acids R17, N18 and R39 (right). **(D)** Bar chart of percentage of unique target genes with at least one FruM consensus motif in the 1kbp enhancer sequence upstream of translational start site for genes in s_142_ or non-differentially expressed genes between *fruM* male and control male pedicels (non-DEG group). Only identified motifs that showed conservation at positions 2, 3 and 5 with the consensus motif were included for analysis. * *p* < 0.05, Chi-squared test. The data underlying this figure can be found at https://doi.org/10.17632/dtscz72px6.1.
(TIF)

S1 Table Statistical values for comparisons between groups for summed number of beam breaks during ZT11–ZT12. Data collected from Locomotor Activity Monitor assays. Pairwise Wilcoxon tests with Benjamini–Hochberg correction used for comparisons.
(XLSX)

S2 Table Statistical values for r_50_ comparisons between groups for distance from center of cage for BuzzWatch recordings. Data collected from BuzzWatch recordings. Pairwise Wilcoxon tests with Benjamini–Hochberg correction used for comparisons.
(XLSX)

S3 Table Statistical values for comparisons between groups for fly-by counts at dusk. Data collected from Fly-by recordings. Pairwise *t*-tests with Benjamini–Hochberg correction used for comparisons.
(XLSX)

S4 Table Statistical values for comparisons between groups for Wing Beat Frequency. Data collected from Fly-by recordings. Pairwise *t*-tests with Benjamini–Hochberg correction used for comparisons.
(XLSX)

S5 Table Statistical values for comparisons between groups for mechanical tuning frequency during White Noise playback. Data collected using laser Doppler vibrometry during white noise stimulus playback. Pairwise *t*-tests with Benjamini–Hochberg correction used for comparisons.
(XLSX)

S6 Table Statistical values for comparisons between groups for mechanical tuning frequency in unstimulated, sedated state. Data collected using laser Doppler vibrometry from unstimulated, sedated mosquito ears. Pairwise *t*-tests with Benjamini–Hochberg correction used for comparisons.
(XLSX)

S7 Table Statistical values for comparisons between groups for differences in mechanical tuning frequency in stimulated, active state and unstimulated, sedated state. Data collected using laser Doppler vibrometry. Pairwise *t*-tests with Benjamini–Hochberg correction used for comparisons.
(XLSX)

S8 Table Statistical values for comparisons between groups for mechanical tuning frequency during sweep stimulation. Data collected using combined vibrometry and electrophysiology paradigm utilizing sweep stimuli. Pairwise *t*-tests with Benjamini–Hochberg correction used for comparisons.
(XLSX)

S9 Table Statistical values for comparisons between groups for electrical tuning frequency during sweep stimulation. Data collected using combined vibrometry and electrophysiology paradigm utilizing sweep stimuli. Pairwise *t*-tests with Benjamini–Hochberg correction used for comparisons.
(XLSX)

S10 Table Statistical values for comparisons between groups for threshold minima electrical tuning frequency during pure tone stimulation. Data collected using combined vibrometry and electrophysiology paradigm utilizing pure tone stimuli. Pairwise Wilcoxon tests with Benjamini–Hochberg correction used for comparisons.
(XLSX)

S11 Table Statistical values for comparisons between groups for minimum voltage required to stimulate a significant compound action potential during pure tone stimulation. Data collected using combined vibrometry and electrophysiology paradigm utilizing pure tone stimuli. Pairwise Wilcoxon tests with Benjamini–Hochberg correction used for comparisons.
(XLSX)

S12 Table Statistical values for comparisons between groups for maximum action potential responses during pure tone stimulation. Data collected using combined vibrometry and electrophysiology paradigm utilizing pure tone stimuli. Pairwise Wilcoxon tests with Benjamini–Hochberg correction used for comparisons.
(XLSX)

S13 Table Statistical values for comparisons between groups for maximum action potential responses during step stimulation. Data collected using combined vibrometry and electrophysiology paradigm utilizing step (static deflection) stimuli. Pairwise *t*-tests with Benjamini–Hochberg correction used for comparisons.
(XLSX)

S14 Table JO width measurements. All values provided as means ± standard deviations. Numbers in brackets refer to sample sizes for each experiment.
(XLSX)

S15 Table Statistical values for comparisons between groups for JO width measurements. Pairwise *t*-tests with Benjamini–Hochberg correction used for comparisons.
(XLSX)

S16 Table Median values of maximum AUC ratio for Ringer and TeNT injection datasets (vibrometry). All values provided as medians ± standard errors. Numbers in brackets refer to sample sizes for each experiment.
(XLSX)

S17 Table Median values of maximum Δ frequency and AUC ratio for Ringer and cAMP injection datasets (vibrometry). All values provided as medians ± standard errors. Numbers in brackets refer to sample sizes for each experiment.
(XLSX)

S18 Table Statistical values for maximum AUC ratio comparisons for Ringer and cAMP injection datasets. Data collected using laser Doppler vibrometry coupled with injections. Wilcoxon tests were used for comparisons.
(XLSX)

S19 Table Statistical values for maximum Δ frequency comparisons for Ringer and cAMP injection datasets. Data collected using laser Doppler vibrometry coupled with injections. Wilcoxon tests were used for comparisons.
(XLSX)

S20 Table Normalized counts of pedicel RNAsequencing data. DESeq2 normalized counts for control female, control male and *fruM* male pedicel RNAsequencing data.
(XLSX)

S21 Table Comparisons of control female and male pedicel RNAsequencing data. DESeq2 output for comparisons between control female and control male pedicel RNAsequencing data.
(XLSX)

S22 Table Comparisons of *fruM* male and control female pedicel RNAsequencing data. DESeq2 output for comparisons between *fruM* male and control female pedicel RNAsequencing data.
(XLSX)

S23 Table Comparisons of *fruM* male and control male pedicel RNAsequencing data. DESeq2 output for comparisons between *fruM* male and control male pedicel RNAsequencing data.
(XLSX)

S24 Table Motif scanning results for genes in s_142_. Output of HOMER FruM core consensus motif (7 bp) scanning results for genes in s_142_.
(XLSX)

S25 Table Motif scanning results for non-DEG group genes. Output of HOMER FruM core consensus motif (7 bp) scanning results for genes not differentially expressed between *fruM* male and control male pedicels (non-DEG group).
(XLSX)

S1 Raw Images Gel images of EMSA data. Original gel images of EMSA data shown in Fig 5H. Protein-DNA interactions were examined between zinc-finger domain (Zn-B) of FruMB (left) or truncated FruMB without the zinc-finger domain (right) and the two 100 bp enhancer fragments of AAEL006647 that each bears a centered putative FruM-binding consensus DNA motif (see Figs 5B and 5G). Interactions represented as band shifts were observed only between Zn-B domain and the two 100 bp enhancer fragments.
(PDF)

S1 Video Mosquito flight activity. Flight activity of groups of control female, control male and *fruM* male mosquitoes during ZT11.5–ZT12. Thirty mosquitoes per cage entrained for three days prior to recording. Video available at https://doi.org/10.17632/dtscz72px6.1.
(MP4)

S2 Video Representative flight trajectory tracking video. Representative video of tracking of *fruM* male flight trajectories using BuzzWatch system. Video available at https://doi.org/10.17632/dtscz72px6.1.
(MP4)

S3 Video Control and *fruM* male responses to 400 Hz tone. Control male and *fruM* male responses to playback of 400 Hz tone. Right container houses 4 *fruM* males, left container 4 control males. Video available at https://doi.org/10.17632/dtscz72px6.1.
(MP4)

S4 Video Control and *fruM* male responses to 450 Hz tone. Control male and *fruM* male responses to playback of 450 Hz tone. Right container houses 4 *fruM* males, left container 4 control males. Video available at https://doi.org/10.17632/dtscz72px6.1.
(MP4)

S5 Video Control and *fruM* male responses to 500 Hz tone. Control male and *fruM* male responses to playback of 500 Hz tone. Right container houses 4 *fruM* males, left container 4 control males. Video available at https://doi.org/10.17632/dtscz72px6.1.
(MP4)

S6 Video Control and *fruM* male responses to 550 Hz tone. Control male and *fruM* male responses to playback of 550 Hz tone. Right container houses 4 *fruM* males, left container 4 control males. Video available at https://doi.org/10.17632/dtscz72px6.1.
(MP4)

S7 Video Control and *fruM* male responses to 470 Hz tone. Control male and *fruM* male responses to playback of 470 Hz tone. Left container houses 4 *fruM* males, right container 4 control males. Video available at https://doi.org/10.17632/dtscz72px6.1.
(MP4)

## Abbreviations

AL: antennal lobe
AMMC: antennal mechanosensory and motor center
AUC: Area Under the Curve
BH: Benjamini–Hochberg
CNS: central nervous system
DSHB: Developmental Studies Hybridoma Bank
EMSA: Electrophoretic Mobility Shift Assay
GO: Gene ontology
JO: Johnston’s Organ
LD: Light:Dark
NGS: normal goat serum
PBS: phosphate-buffered saline
PCA: Principal Component Analysis
PVL: Particle Velocity Level
SOs: self-sustained oscillations
SPL: Sound Pressure Level
TeNT: tetanus toxin
TFs: transcription factors
TSS: translational start sites
WBF: Wing Beat Frequency
ZT: Zeitgeber Time
