# Supplementary material for: fruitless is essential for male Aedes aegypti mosquito attraction to female flight sounds
Source: PLoS Biol. 2026 Oct 27;24(10):e3004041. doi: 10.1371/journal.pbio.3004041 (PMC13712528; doi:10.1371/journal.pbio.3004041)

FruMB Zinc Finger

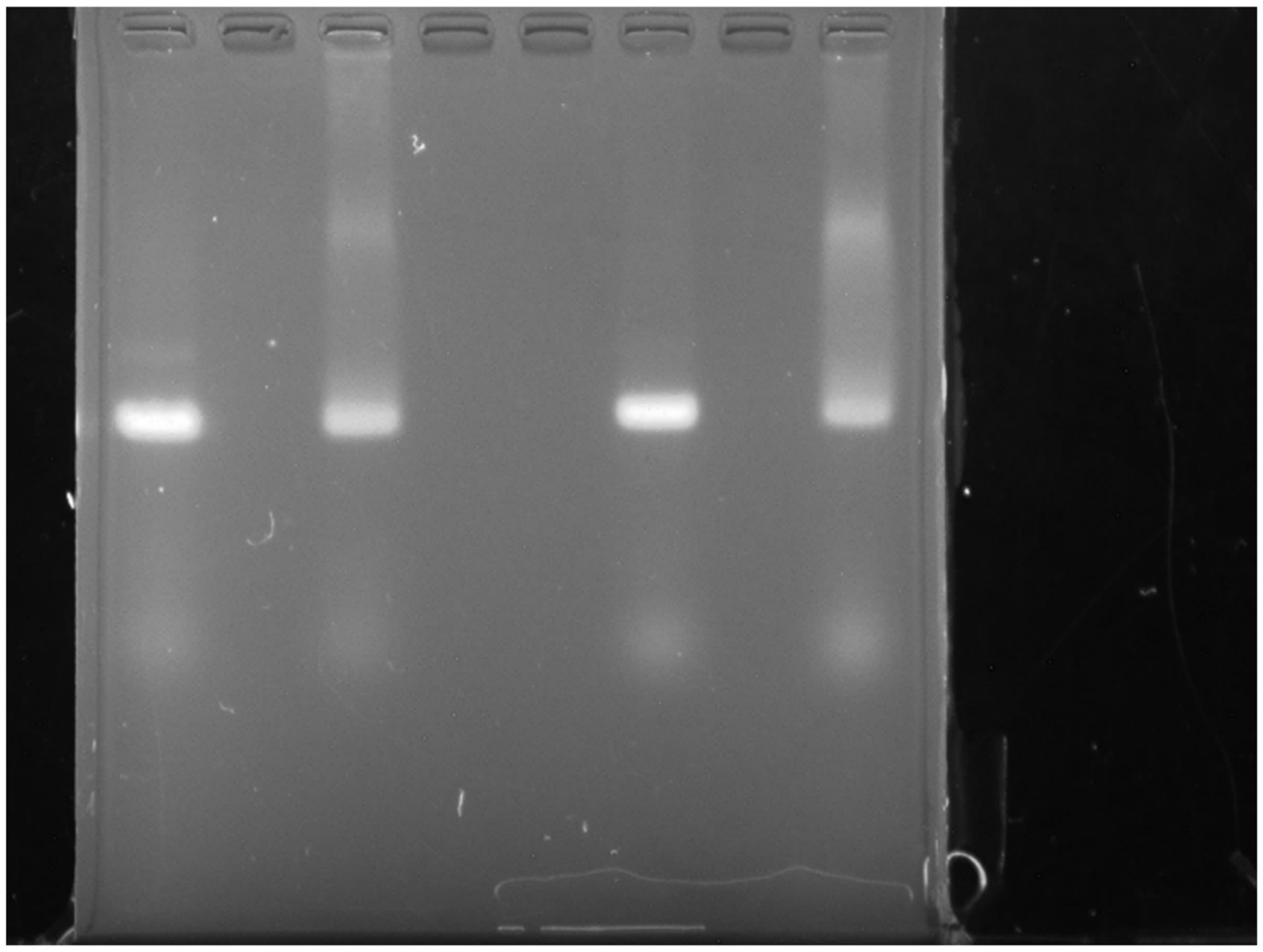

Truncated FruMB

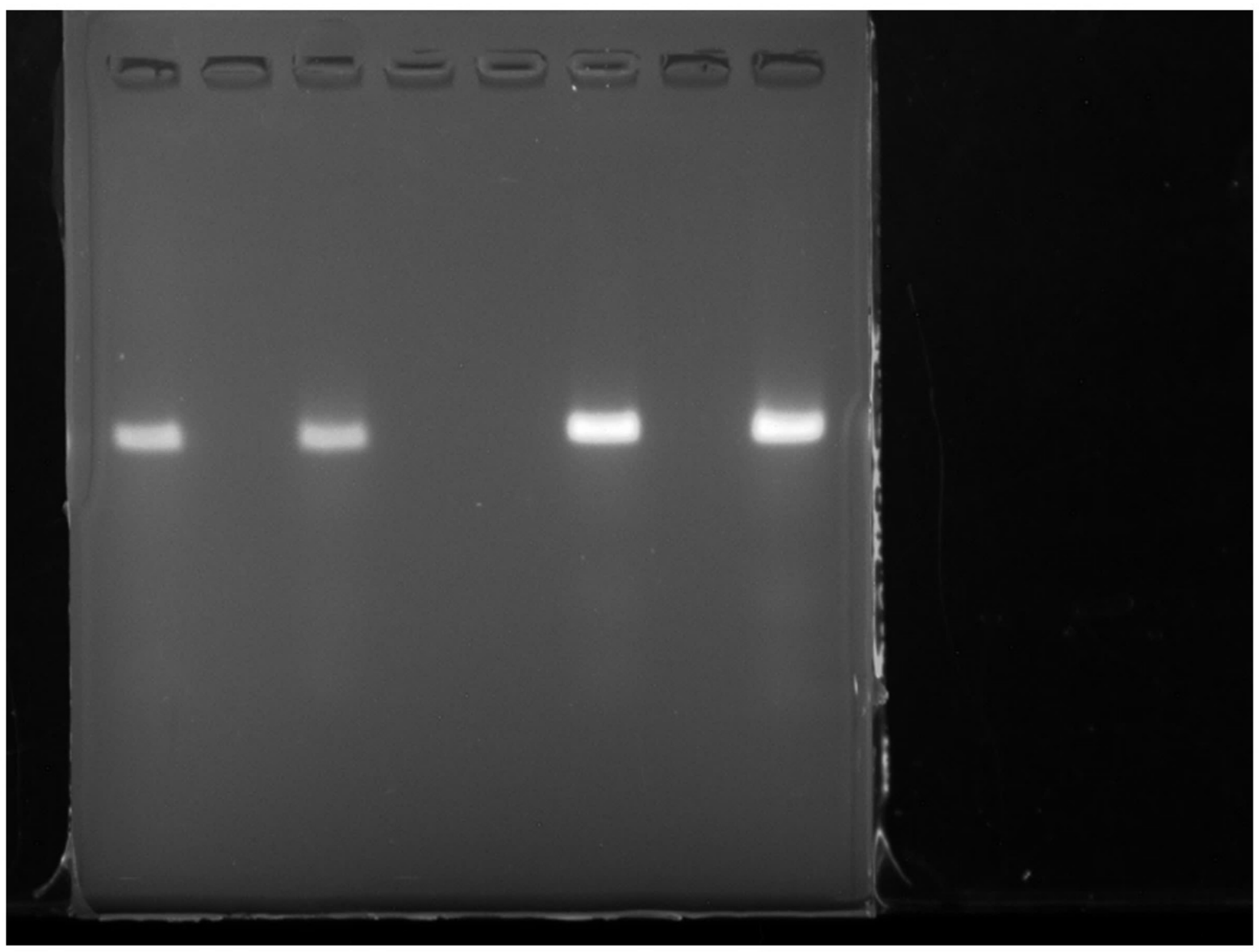

Supplement: Supplementary file 1 [file pbio.3004041.s047.pdf]
